# Dataset of parenting practices, self-control and anti-social behaviors: Meta-analytic structural equation modeling

**DOI:** 10.1016/j.dib.2020.106114

**Published:** 2020-08-04

**Authors:** Hossein Dabiriyan Tehrani, Sara Yamini

**Affiliations:** a Ph.D in sociology, university of Allameh Tabataba'i, Tehran, Iran; b Faculty of management, Kar University, Tehran, Iran

**Keywords:** General theory of crime, Parenting, Self-control, Meta-analytic structural equation modeling, Two-stage meta-analytic structural equation modeling, One stage meta-analytic structural equation modelin

## Abstract

This dataset is used to clarify the nexus between effective parenting practices, low self-control, and anti-social behaviors in Gottfredson and Hirschi's General Theory of Crime (GTC). The analysis included 72 articles reporting 255 effect sizes (N = 94,604). We used the method of Meta-Analytic Structural Equation Modeling (MASEM) to test the assumptions of GTC. In this regard, we employed Two-Stage Meta-Analytic Structural Equation Modeling (TSSEM) and One Stage Meta-Analytic Structural Equation Modeling (OSMASEM) to perform MASEM and its moderators. The findings of the MASEM revealed that low self-control is a positive and in magnitude modest determinant of anti-social behaviors. The effective parenting practice is negative, of small size, and also a statistically significant determinant of low self-control. We observed that effective parenting practice is statistically significant and, in magnitude, shows small size negative direct and indirect effects on anti-social behaviors. That is, low self-control partially mediated the relationship between effective parenting practices and anti-social behaviors. Consistent with the construct of aggregated effective parenting practices, we found uniform patterns for models performed across the elements of effective parenting practices (i.e., emotionally supportive practices, monitoring, recognition, and effective discipline) with low self-control and anti-social behaviors. The findings of moderator analyses showed that the association between low self-control and anti-social behaviors tended to be stronger when the individualistic score of countries improved.

Specifications Table

**Table:** 

Subject	Social Sciences (General), Law, Social Psychology
Specific subject area	Criminology, Parenting practices, Self-control, Anti-social behavior
Type of data	4 Tables, 3 Figures
How data were acquired	The electronic search
Data format	Analyzed secondary data
Parameters for data collection	Only research was included that reported correlation among the constructs of this research quantitatively (i.e., parenting practices, self-control, and anti-social behavior), theoretical reviews, conceptual articles, or qualitative researches were excluded. No restriction regarding the language of publication was applied in the selection of the primary research.
Description of data collection	Data were collected through the electronic search of ProQuest, PsycINFO, Scopus, and Web of Science, and also the American Society of Criminology, National Criminal Justice Reference Service [NCJRS], Criminal Justice Abstracts.
Data source location	Allameh Tabataba'i University
Data accessibility	Dabiriyan Tehrani, Hossein; Yamini, Sara (2020), “Parenting Practices, Self-Control and Anti-Social Behaviors: Meta-Analytic Structural Equation Modeling”, Mendeley Data, v4http://dx.doi.org/10.17632/82vxgs8t7n.4 https://data.mendeley.com/datasets/82vxgs8t7n/4
Related research article	“Parenting Practices, Self-Control and Anti-Social Behaviors: Meta-Analytic Structural Equation Modeling” https://doi.org/10.1016/j.jcrimjus.2020.101687 Received 16 February 2020; Received in revised form 15 April 2020; Accepted 15 April 2020

Value of the data

• This dataset also allows researchers to reproduce this meta-analysis. It permits to re-analyze data with novel statistical techniques that will be developed in the future.
• This dataset will facilitate future updates of this meta-analysis. It contributes to improving the credibility of meta-analytic conclusions and cumulative scientific knowledge.
• Access to the data will make better interpretations of analytical findings that are presented in tables, and figures.
• Detailed datasets, which are made publicly available in an SPSS file attached to this article, will encourage further explorative investigations in this area of research.
• This dataset will be useful for undergraduate students. Because the current meta-analysis to test the assumptions of GTC and bridge the gaps in empirical literature used the state-of-the-art methods of MASEM. In this regard, we used TSSEM to perform MASEM. Inconsistencies were explained by categorical moderator analyses through TSSEM and OSMASEM, and also continuous moderator analyses through OSMASEM. We employed parameter-based MASEM (using bootstrap and delta methods) and Full Information Meta-Analytic Structural Equation Modeling (FIMASEM) to show the generalizability and heterogeneity of Structural Equation Modeling (SEM) parameters. Likewise, this research applied studentized deleted residuals (SDRs) to assess outlier analysis and also conducted different methods (i.e., TSSEM with corrected correlation, Univariate-r MASEM, FIMASEM, OSMASEM) to perform MASEM to check the robustness of the findings resulted from the principal method used in this meta-analysis (i.e., TSSEM). Finally, we used multiple methods of assessing for publication bias, namely, funnel plot, trim and fill analysis, Fail-safe N, and Egger's test. Accordingly, the reproducibility of this research based on this dataset enables researchers to enhance their methodological knowledge on different methods of MASEM.

## 1 Data description

In order to acquire the data, we conducted the electronic search through ProQuest, PsycINFO, Scopus, and Web of Science, and also the American Society of Criminology, National Criminal Justice Reference Service [NCJRS], Criminal Justice Abstracts. Because the GTC was introduced in 1990, the time frame of this research ranged from 1 January 1990 to 23 September 2019 for all published and non-published research. Only investigations were included that reported correlation among the constructs of this research quantitatively (i.e., parenting practices, self-control, and anti-social behavior), theoretical reviews, conceptual articles, or qualitative researches were excluded. No restriction regarding the language of publication was applied in the selection of the primary investigations. Based on the investigations included, the following data were entered into this MASEM: the correlations between constructs, the relevant sample sizes, year of publication, the mean age of the sample, individualism score for each country, the proportion of females, kinds of anti-social behavior, mode of assessment, self-control measurements, data extracted from which kind of design (cross-section vs. cross-section made through longitudinal research), the reliability of constructs. The data are summarized through four tables and three figures. Table 1 presented literature review on fully or partially role of low self-control between parenting practices and anti-social behaviors. Table 2 represented list of parenting practices elements and variables. Table 3 showed coded research characteristics used in analysis. Table 4 demonstrated the characteristics of research included in the meta-analysis of “Parenting Practices, Self-Control and Anti-Social Behaviors: Meta-Analytic Structural Equation Modeling”. The Figures of the funnel plot were employed to estimate publication bias between effective parenting practices and low self-control, effective parenting practices and anti-social behavior, low self-control, and anti-social behavior, respectively.

**Table 1:** Literature review on fully or partially role of low self-control between parenting practices and anti-social behaviors.

	Researchers	Elements of parenting practices	Kinds of anti-social behavior
Full mediation model	Jo & Zhang (2014)	AEPP	AASB
	Feldman & A. Weinberger (1994)	AEPP	AASB
	Gibbs et al., (1998)	AEPP	AASB
	Boisvert et al., (2012)	Attachment	AASB
	Cochran et al., [4]	AEPP	Academic dishonesty
	E. Higgins (2002)	AEPP	AASB
	Simons el al (2007)	Monitoring/Discipline	AASB
		Supportive involvement	
		Hostility/Rejection	
Partial mediation model	Muftić et al., (2014)	AEPP	Violence perpetration
		AEPP	Property offending
	C. Lagrange (1999)	Supervision	Violent offenses
	Hay (2001)	Monitoring/Discipline	AASB
	Gibbs et al., [7]	AEPP	AASB
	Vazsonyi & Belliston (2007)	Support	AASB
		Monitoring	AASB
	Benda (2003)	AEPP	AASB
	Chapple et al., (2005)	Monitoring	Substance Use
	Finkenauer et al., (2005)	AEPP	Behavioral problems
	Jones et al.,(2007)	Support	AASB
	Kort-Butler et al., (2011)	Monitoring	Criminal behavior
	Morris et al., (2007)	AEPP	AASB
	Perrone et al., (2004)	AEPP	AASB
	Unnever et al.,(2003)	AEPP	AASB
	Boisvert et al., (2012)	Rejection	AASB

*Note*. AEPP is Aggregated construct of Effective Parenting Practices; AASB is Aggregated construct of Anti-Social Behavior.

**Table 2:** List of parenting practices.

The element of parenting practices	Parenting behaviors	Names and words in description
Emotionally supportive practices		
	Affection	Warmth
		Affection
		Acceptance
		Affective tie
		Hugs
		Loving
		Positive feelings
		Smiles
		Intimate relationship
	Support	Emotional support
		Understanding
		Helpful
		Encouraging
		Trust
	Closeness	Involvement
		Cohesion
		Attachment
		Attention
		Pay attention
		Care
	Neglectful (−)	Neglect
		Avoidance
	Rejection (−)	Rejection
		Conflict
		Withdrawal
	Hostility (−)	Hostility
		Anger
		Annoyance
		Irritation
		Sarcasm
Monitoring		
	Supervision	Tracking of activities
		Tracking of whereabouts
		Tracking the child's behavior
		Checking homework
		Awareness of activities
Recognition		
	Recognize the anti-social behaviors	The ability of the parent to recognize when youth engage in anti-social behaviors
Effective discipline		
	Fair and Non-corporal means of punishment	Calmly discuss misbehavior
		Noticing when doing good
		Withdrawal of privileges
		Consistent discipline
		Proportionate punishment
		Agree on discipline
		Responsive discipline
	Harsh discipline (−)	Firm control
		Harsh punishment
	Physical punishment (−)	Beaten child up
		Hitting
		Kicking
		Slapping
	Verbal aggression as punishment (−)	Abusive name calling
		Yelling
		Nagging
		Scolding
		Verbal attacks
		Threatening to hit

*Note.* This research equalized the direction of effect sizes (multiplied by −1 as needed), for neglectful, rejection, hostility, harsh discipline, verbal aggression, and physical punishment as the different manifestations of parenting practices’ elements, to display relationships of effective parenting practices with low self-control and anti-social behaviors.

**Table 3:** Coded research characteristics used in analysis.

Characteristic	Coded as	Used to
Sample size (n)	(C) Number of participants included in the analysis	Weight each research findings
Sex (female)	(C) Proportion of female respondents	Proportion of female, mean age and culture used as a continues moderators on the relationship of effective parenting practices, low self-control, and anti-social behavior.
Age	(C) Mean age of the sample	
Culture	(C) Hofstede's individualism score	
Mode of assessment	(CA)The perspective from which participants' low self-control and anti-social behaviors were assessed. Due to a small number of alternative categories coded as self-report (1) vs other (0).	
Type of anti-social behaviors	(CA) vandalism, theft, and assault, group fight, shot or stabbed someone, and pulled a knife or a gun on someone, physical assault, shoplifting, carry a hidden weapon, attack someone categories coded as crime (1) Alcohol use, school misconduct, sell drugs, write bad checks, gang membership, nonviolent crime, substances use, childhood antisociality, risky lifestyles, running away home, risk-taking behaviors categories coded as analogous behavior (2), and general deviance (3).	
Data extracted from which kinds of design	(CA) Data extracted from cross-sectional investigations versus cross-section made through longitudinal investigations. Categories coded as cross-section (1) and cross-section made through longitudinal investigations (2).	
Self-control measurements	(CA) Due to a small number of alternative categories coded as Grasmick (1993) constructed a 24-item (1) and other measurements (2).	

*Note*. (C) = continuous, (CA) = categorical variables.

**Table 4:** Characteristics of included research.

Author name	Sample		Mean	IND	Kinds		LSC	Kinds of	Alpha	Alpha	Alpha
(Year) (Country)	Size	Female%	age	score	ASB	MODEASS	MEAS	design	Parenting	LSC	ASB
(Vazsonyi et al., 2016) (Czech)											
Setting 1	239	47.5	14.02	58	AB	SR	GR93	CS	.71	.83	.88
Setting 2	239	47.5	14.02	58	AB	SR	GR93	CS	.71	.83	.67
Setting 3	239	47.5	14.02	58	CR	SR	GR93	CS	.71	.83	.76
Setting 4	130	47.7	14.71	58	AB	SR	GR93	CS	.79	.82	.88
Setting 5	130	47.7	14.71	58	AB	SR	GR93	CS	.79	.82	.70
Setting 6	130	47.7	14.71	58	CR	SR	GR93	CS	.79	.82	.87
(Alvarez-Rivera & Fox, 2010) (Puerto Rico)	298	54	16.23	27	AB	SR	GR93	CS	.92	.68	.82
(Anderson *et al.*, 2015) (U.S.)											
Setting 1	1072	51.4	13.53	91	AB	SR	OTHER	LON	.72	.65	.94
Setting 2	1072	51.4	13.53	91	CR	SR	OTHER	LON	.72	.65	.86
Setting 3	1072	51.4	13.53	91	CR	SR	OTHER	LON	.72	.65	
(Baker, 2010) (U.S.)	4834	52	16.02	91	AB	OTHER	OTHER	LON	.78	.68	
(Bobbio *et al.*, 2019) (Argentina)	214	00	15.89	46	CR	SR	GR93	CS	.8	.85	.76
(Boccio & Beaver, 2018)(U.S.)											
Setting 1	346	50	15.5	91	CR	OTHER	OTHER	LON	.55	.66	.6
Setting 2	346	50	15.5	91	CR	OTHER	OTHER	LON	.64	.66	.6
Setting 3	346	50	15.5	91	CR	OTHER	OTHER	LON	.84	.66	.6
Setting 4	346	50	15.5	91	AB	OTHER	OTHER	LON	.55	.66	.53
Setting 5	346	50	15.5	91	AB	OTHER	OTHER	LON	.64	.66	.53
Setting 6	346	50	15.5	91	AB	OTHER	OTHER	LON	.84	.66	.53
(Brownfield, 2010) (Canada)	618	52.8	14	80	AB	SR	OTHER	CS			
(Burt & Ronald, 2006) (U.S.)	754	53	13	91	CR	OTHER	GR93	LON	.77	.89	.9
(Cheung & Cheung, 2008) (Hong Kong)											
Setting 1	1015	54	16.01	25	AB	SR	OTHER	CS	.66	.61	.73
Setting 2	1015	54	16.01	25	CR	SR	OTHER	CS	.66	.61	.5
Setting 3	1015	54	16.01	25	CR	SR	OTHER	CS	.66	.61	.5
Setting 4	1015	54	16.01	25	CR	SR	OTHER	CS	.56	.61	.73
Setting 5	1015	54	16.01	25	CR	SR	OTHER	CS	.56	.61	.5
Setting 6	1015	54	16.01	25	CR	SR	OTHER	CS	.56	.61	.5
(Cheung & Cheung, 2010) (Hong Kong)											
Setting 1	1015	54	16	25	CR	SR	OTHER	CS	.66	.61	.79
Setting 2	1015	54	16	25	CR	SR	OTHER	CS	.56	.61	.7
(Costello & Dunaway, 2003) (U.S.)											
Setting 1	377	52.51	15	91	CR	SR	GR93	CS	.84	.78	.64
Setting 2	377	52.51	15	91	AB	SR	GR93	CS	.84	.78	.84
(Evans *et al.*, 2012) (U.S.)											
Setting 1	381	100	13	91	GC	SR	OTHER	LON	.77	.73	.9
Setting 2	381	100	13	91	GC	SR	OTHER	LON	.70	.73	.9
Setting 3	381	100	13	91	GC	SR	OTHER	LON	.88	.73	.9
Setting 4	381	100	13	91	GC	SR	OTHER	LON	.71	.73	.9
Setting 5	381	0	13	91	GC	SR	OTHER	LON	.77	.73	.9
Setting 6	381	0	13	91	GC	SR	OTHER	LON	.70	.73	.9
Setting 7	381	0	13	91	GC	SR	OTHER	LON	.88	.73	.9
Setting 8	381	0	13	91	GC	SR	OTHER	LON	.71	.73	.9
(Frijns *et al.*,2005) (Netherlands)	1173	49	12.3	80	GC	SR	OTHER	LON	.79	.7	.93
(Guo, 2018)(U.S.)	1020	50	12.23	91	GC	SR	OTHER	CS	.68	.83	.65
(Hay, 2001) (U.S.)											
Setting 1	197	50	16	91	AB	SR	GR93	CS	.79	.81	.45
Setting 2	197	50	16	91	AB	SR	GR93	CS	.45	.81	.45
Setting 3	197	50	16	91	AB	SR	GR93	CS	.88	.81	.45
Setting 4	197	50	16	91	AB	SR	GR93	CS	.77	.81	.45
Setting 5	197	50	16	91	AB	SR	GR93	CS	.85	.81	.45
Setting 6	197	50	16	91	AB	SR	GR93	CS	.79	.81	.61
Setting 7	197	50	16	91	AB	SR	GR93	CS	.45	.81	.61
Setting 8	197	50	16	91	AB	SR	GR93	CS	.88	.81	.61
Setting 9	197	50	16	91	AB	SR	GR93	CS	.77	.81	.61
Setting 10	197	50	16	91	AB	SR	GR93	CS	.85	.81	.61
(Hay & Forrest, 2008) (U.S.)	750	52	13.22	91	GC	SR	OTHER	CS	.79	.63	
(Higgins, 2002) (U.S.)	425	52.9	21	91	GC	SR	OTHER	CS	.92	.91	.8
(Huang, 2007) (U.S.)	985	50.6	11	91	AB	OTHER	OTHER	LON	.7	.8	.8
(Intravia *et al.*, 2012) (U.S.)	1675	50	13.79	91	CR	SR	OTHER	CS			.84
(Schreck *et al.*, 2002) (U.S.)	1101	51	15.5	91	AB	SR	GR93	CS			.84
(Unnever et al., 2006) (U.S.)											
Setting 1	2472	51.1	12.39	91	AB	SR	GR93	CS	.77	.87	.78
Setting 2	2472	51.1	12.39	91	AB	SR	GR93	CS	.74	.87	.78
Setting 3	2472	51.1	12.39	91	AB	SR	GR93	CS		.87	.78
Setting 4	2472	51.1	12.39	91	AB	SR	GR93	CS	.72	.87	.78
Setting 5	2472	51.1	12.39	91	AB	SR	GR93	CS	.77	.87	.82
Setting 6	2472	51.1	12.39	91	AB	SR	GR93	CS	.74	.87	.82
Setting 7	2472	51.1	12.39	91	AB	SR	GR93	CS		.87	.82
Setting 8	2472	51.1	12.39	91	AB	SR	GR93	CS	.72	.87	.8
(Janssen et al., 2016) (Netherlands)	615	48	13.9	80	GC	SR	GR93	LON	.82	.72	.83
(Janssen et al., 2017) (Netherlands)											
Setting 1	315	00	13	80	GC	SR	GR93	LON	.77	.75	.85
Setting 2	288	100	13	80	GC	SR	GR93	LON	.77	.75	.85
(Jennings et al., 2010) (U.S.)	407	58.3	16	91	AB	SR	OTHER	CS	.75	.83	
(Jeon & Chun, 2017) (South Korea)											
Setting 1	3449	50	14	18	AB	SR	OTHER	CS	.86	.65	.54
Setting 2	3449	50	14	18	AB	SR	OTHER	CS	.86	.65	.64
(Youngoh Jo & Lee, 2018) (South Korea)	2491	85.3	11	18	CR	SR	GR93	LON	.82	.64	.55
(Junger & Tremblay, 1999) (Canada)											
Setting 1	731	00	13.5	80	CR	SR	GR93	LON	.81	.85	.92
Setting 2	731	00	13.5	80	CR	SR	GR93	LON	.81	.85	.79
(Kazemian et al., 2009) (Canada)	470	00	16.9	80	AB	OTHER	OTHER	LON	.68	.75	.62
(Kuhn & Laird, 2013) (U.S.)											
Setting 1	180	51	12.04	91	AB	SR	GR93	CS	.79	.88	.87
Setting 2	180	51	12.04	91	AB	SR	GR93	CS	.58	.88	.87
(Li et al., 2019) (Poland)											
Setting 1	146	00	16.97	60	AB	SR	OTHER	CS	.95	.77	.77
Setting 2	146	00	16.97	60	AB	SR	OTHER	CS	.91	.77	.77
Setting 3	355	00	16.97	60	AB	SR	OTHER	CS	.95	.77	.77
Setting 4	355	00	16.97	60	AB	SR	OTHER	CS	.91	.77	.77
(Longshore et al., 2005) (U.S.)	359	26	16	91	CR	SR	OTHER	LON	.51	.54	.58
(Kort-Butler et al., 2011) (U.S.)											
Setting 1	199	40	21.5	91	CR	OTHER	GR93	CS	.75	.82	.89
Setting 2	199	40	21.5	91	CR	OTHER	GR93	CS	.88	.82	.89
Setting 3	199	40	21.5	91	CR	OTHER	GR93	CS	.83	.82	.89
Setting 4	199	40	21.5	91	CR	OTHER	GR93	CS	.75	.82	.78
Setting 5	199	40	21.5	91	CR	OTHER	GR93	CS	.88	.82	.78
Setting 6	199	40	21.5	91	CR	OTHER	GR93	CS	.83	.82	.78
(McGloin et al., 2004) (U.S.)	1725	49	12.73	91	AB	OTHER	GR93	LON		.78	.85
(McKee, 2012) (U.S.)	1409	54	12.2	91	GC	SR	GR93	LON	.72	.7	.85
(Meldrum, 2008) (U.S.)	1034	48	12.07	91	CR	OTHER	OTHER	LON		.64	
(Meldrum et al., 2013) (U.S.)	825	50	15	91	GC	SR	OTHER	LON	.91	.82	.82
(Meldrum et al., 2015) (U.S.)											
Setting 1	101	22	15.67	91	AB	OTHER	GR93	CS	.85	.92	
Setting 2	101	22	15.67	91	AB	OTHER	GR93	CS	.89	.92	
Setting 3	101	22	15.67	91	AB	OTHER	GR93	CS	.88	.92	
(Meldrum, et al., 2009) (U.S.)	1364	46	14.03	91	AB	SR	GR93	LON	.8	.78	.61
(Miller, 2012) (U.S.)											
Setting 1	763	49	11.93	91	AB	SR	OTHER	LON	.69	.68	
Setting 2	763	49	11.93	91	CR	SR	OTHER	LON	.69	.68	
(Moon & Morash, 2013) (U.S.)											
Setting 1	296	57	14	91	CR	SR	GR93	CS	.65	.9	.91
Setting 2	296	57	14	91	CR	SR	GR93	CS	.84	.9	.91
Setting 3	296	57	14	91	CR	SR	GR93	CS	.85	.9	.91
Setting 4	296	57	14	91	GC	SR	GR93	CS	.65	.9	.79
Setting 5	296	57	14	91	GC	SR	GR93	CS	.85	.9	.79
Setting 6	296	57	14	91	GC	SR	GR93	CS	.85	.9	.79
Setting 7	296	57	14	91	CR	SR	GR93	CS	.65	.9	.82
Setting 8	296	57	14	91	GC	SR	GR93	CS	.84	.9	.82
Setting 9	296	57	14	91	GC	SR	GR93	CS	.85	.9	.79
(Moon & Alarid, 2015) (U.S.)	296	57	14	91	GC	SR	GR93	CS	.9	.9	.88
(Muftić et al., 2014) (U.S.)	1759	50.50	13.79	91	GC	SR	GR93	LON		.85	.73
(Kabiri et al., 2019) (Iran)	784	44	24.3	41	AB	SR	GR93	CS	.88	.88	.89
(Schreck, 2002) (U.S.)	1054	51	16	91	CR	SR	GR93	CS			
(Simons et al., 2007) (U.S.)											
Setting 1	867	54	10.5	91	AB	SR	OTHER	LON	.75	.8	
Setting 2	867	54	10.5	91	AB	SR	OTHER	LON	.83	.8	
Setting 3	867	54	10.5	91	AB	SR	OTHER	LON	.79	.8	
(Vazsonyi et al., 2007) (Hungry)											
Setting 1	826	31.6	16.6	80	GC	SR	GR93	CS	.79	.83	.96
Setting 2	826	31.6	16.6	80	GC	SR	GR93	CS	.70	.83	.96
Setting 3	826	31.6	16.6	80	GC	SR	GR93	CS	.75	.83	.96
(Vazsonyi & Belliston, 2007) (Japan)											
Setting 1	344	66.6	19.8	46	GC	SR	GR93	CS	.75	.8	.91
Setting 2	344	66.6	19.8	46	GC	SR	GR93	CS	.74	.8	.91
Setting 3	344	66.6	19.8	46	GC	SR	GR93	CS	.69	.8	.91
(Vazsonyi & Belliston, 2007) (Netherland)											
Setting 1	1244	53.3	16.10	80	GC	SR	GR93	CS	.74	.85	.95
Setting 2	1244	53.3	16.10	80	GC	SR	GR93	CS	.73	.85	.95
Setting 3	1244	53.3	16.10	80	GC	SR	GR93	CS	.72	.85	.95
(Vazsonyi & Belliston, 2007) (Switzerland)											
Setting 1	3819	37.5	18.2	68	GC	SR	GR93	CS	.74	.8	.96
Setting 2	3819	37.5	18.2	68	GC	SR	GR93	CS	.75	.8	.96
Setting 3	3819	37.5	18.2	68	GC	SR	GR93	CS	.79	.8	.96
(Vazsonyi & Belliston, 2007) (U.S.)											
Setting 1	1273	61.4	20	91	GC	SR	GR93	CS	.87	.85	.95
Setting 2	1273	61.4	20	91	GC	SR	GR93	CS	.83	.85	.95
Setting 3	1273	61.4	20	91	GC	SR	GR93	CS	.79	.85	.95
(Vazsonyi & Belliston, 2007) (U.S.)											
Setting 1	802	49.9	16.4	91	GC	SR	GR93	CS	.84	.91	.97
Setting 2	802	49.9	16.4	91	GC	SR	GR93	CS	.83	.91	.97
Setting 3	802	49.9	16.4	91	GC	SR	GR93	CS	.78	.91	.97
(Vazsonyi & Belliston, 2007) (U.S.)											
Setting 1	689	53.6	15.7	91	GC	SR	GR93	CS	.88	.92	.99
Setting 2	689	53.6	15.7	91	GC	SR	GR93	CS	.83	.92	.99
Setting 3	689	53.6	15.7	91	GC	SR	GR93	CS	.85	.92	.99
(Vazsonyi & Klanjšek, 2008) (Switzerland)											
Setting 1	2603	29.5	18.22	68	GC	SR	GR93	CS	.74	.8	.96
Setting 2	2603	29.5	18.22	68	GC	SR	GR93	CS	.76	.8	.96
Setting 3	2603	29.5	18.22	68	GC	SR	GR93	CS	.78	.8	.96
Setting 4	2603	29.5	18.22	68	GC	SR	GR93	CS	.81	.8	.96
Setting 5	2603	29.5	18.22	68	GC	SR	GR93	CS	.76	.8	.96
Setting 6	2603	29.5	18.22	68	GC	SR	GR93	CS	.85	.8	.96
Setting 7	2603	29.5	18.22	68	GC	SR	GR93	CS	.73	.94	.94
Setting 8	2603	29.5	18.22	68	GC	SR	GR93	CS	.71	.94	.94
Setting 9	2603	29.5	18.22	68	GC	SR	GR93	CS	.8	.94	.94
Setting 10	2603	29.5	18.22	68	GC	SR	GR93	CS	.77	.94	.94
Setting 11	2603	29.5	18.22	68	GC	SR	GR93	CS	.86	.94	.94
(Vazsonyi & Huang, 2010) (U.S.)	1364	48.8	10.5	91	GC	SR	OTHER	LON	.73	.81	.8
(Vera & Moon, 2013) (U.S.)											
Setting 1	277	57	14	91	AB	SR	GR93	CS	.84	.9	.94
Setting 2	277	57	14	91	AB	SR	GR93	CS	.84	.9	.94
(Jr et al., 1998) (U.S.)	555	50	40.5	91	GC	SR	GR93	CS	.86	.64	.6
(Wright et al., 2001) (New Zealand)	1037	49	16	79	GC	SR	OTHER	CS			
(You & Kim, 2016) (South Korea)											
Setting 1	448	00	15.2	18	AB	SR	OTHER	CS	.94	.75	.84
Setting 2	282	100	15.2	18	AB	SR	OTHER	CS	.94	.75	.84
(Beaver, 2008) (Canada)											
Setting 1	3780	50	8	80	CR	SR	OTHER	LON	.72	.79	.52
Setting 2	3780	50	8	80	CR	SR	OTHER	LON	.66	.79	.52
Setting 3	3780	50	8	80	CR	SR	OTHER	LON	.57	.79	.52
(Chen, 2017)(China)	600	50	8	20	AB	SR	GR93	CS	.71		.86
(Cho et al., 2005) (South Korea)	2844	46	8	18	AB	SR	2 OTHER	LON	.83	.8	.75
(Finkenauer et al., 2005) (Netherland)											
Setting 1	1359	47.8	12.3	80	CR	SR	OTHER	CS	.8	.67	.84
Setting 2	1359	47.8	12.3	80	CR	SR	OTHER	CS	.65	.67	.84
Setting 3	1359	47.8	12.3	80	CR	SR	OTHER	CS	.68	.67	.84
Setting 4	1359	47.8	12.3	80	CR	SR	OTHER	CS	.8	.67	.68
Setting 5	1359	47.8	12.3	80	CR	SR	OTHER	CS	.65	.67	.68
Setting 6	1359	47.8	12.3	80	CR	SR	OTHER	CS	.68	.67	.68
(Liu et al., 2019) (china)											
Setting 1	917	46.23	14.38	20	AB	SR	OTHER	CS	.94	.88	.71
Setting 2	917	46.23	14.38	20	AB	SR	OTHER	CS	.9	.88	.71
(Marcotte et al., 2002) (Canada)											
Setting 1	249	00	15.09	80	AB	SR	OTHER	CS	.94	.63	.71
Setting 2	279	100	15.09	80	AB	SR	OTHER	CS	.94	.63	.71
(Moon et al., 2012) (South Korea)											
Setting 1	2817	49	14	18	AB	SR	GR93	LON	.87	.63	.67
Setting 2	2817	49	14	18	AB	SR	GR93	LON	.82	.63	.67
(Özdemir et al., 2013) (Turkey)											
Setting 1	546	56.2	16	37	AB	SR	GR93	CS	.77	.83	.8
Setting 2	546	56.2	16	37	AB	SR	GR93	CS	.86	.83	.8
(Shadmanfaat et al., 2018) (Iran)	318	48	23	41	AB	SR	GR93	CS			
[3] (South Korea)	2844	50	13	18	GC	SR	OTHER	CS	.86	.8	
(Watts & McNulty, 2016)(U.S.)	3610	00	16.11	91	GC	OTHER	OTHER	LON	.81	.71	.74
(Wells et al., 2015) (U.S.)	102	00	31.73	91	CR	SR	GR93	CS	.86	.9	.94
(Woeckener et al., 2018) (U.S.)	322	69.25	19.27	91	GC	SR	GR93	CS	.8	.84	.79
(Gelder et al., 2017) (Switzerland)	1197	48	15.04	68	AB	SR	GR93	LON			
(Chae, 2016) (U.S.)	6504	51.61	9.5	91	AB	OTHER	GR93	LON	.84	.69	.73
(Toro, 2010) (U.S.)											
Setting 1	89	61.79	15.58	91	AB	SR	1	CS	.68	.85	
Setting 2	89	61.79	15.58	91	AB	SR	1	CS	.7	.85	
Setting 3	89	61.79	15.58	91	AB	SR	1	CS	.78	.85	
(Morris, 2003) (U.S.)	1504	58.4	17	91	GC	SR	1	CS	.64	.87	.88
(Owens-Sabir, 2005) (U.S.)											
Setting 1	1724	56.6	17.2	91	CR	SR	1	CS	.64	.88	.76
Setting 2	1724	56.6	17.2	91	CR	SR	1	CS	.64	.88	.61
Setting 3	1724	56.6	17.2	91	CR	SR	1	CS	.64	.88	.8
Setting 4	1724	56.6	17.2	91	CR	SR	1	CS	.64	.88	.74
Setting 5	1724	56.6	17.2	91	CR	SR	1	CS	.72	.88	.76
Setting 6	1724	56.6	17.2	91	CR	SR	1	CS	.72	.88	.61
Setting 7	1724	56.6	17.2	91	CR	SR	1	CS	.72	.88	.8
Setting 8	1724	56.6	17.2	91	CR	SR	1	CS	.72	.88	.74
(Brauer, 2011) (U.S.)	1919	50	13.5	91	GC	SR	2	LON		.6	.72

*Note.* IND score=individualism score; Kinds ASB= kinds of anti-social behavior; MODE ASS= mode of assessment; LSC MEAS= Low Self-Control measurement; Alpha_ASB= alpha anti-social behavior Analogous Behavior=AB; Crime= CR; General Crime= GC; Self Report =SR; Cross Section=CS; Longitudinal = LON; Grasmick(1993)= GR93.

## 2 Design, materials, and methods

Two authors scrutinized abstracts and titles of all primary investigations that fulfill the search strategy to determine research eligible for inclusion. Subsequently, two authors independently assessed the full text of potentially relevant non-duplicated investigations. For each research selected for inclusion, the authors separately collected data through a standardized form that was piloted. The inter‐coder reliability of the data coding process was checked by computing the intraclass coefficient of correlation for continuous variables, which yielded an average value of 0.83, and Cohen's kappa coefficient for the categorical variables, which estimated the value of 0.84. The disagreement between authors was resolved by discussion to obtain consensus. Only investigations were included that reported correlation among the constructs of this research quantitatively (i.e., parenting practices, self-control, and anti-social behavior), theoretical reviews, conceptual articles, or qualitative researches were excluded. We developed a detailed coding scheme relying on guidelines recommended by Lipsey and Wilson ([9]), recording research descriptors, and research characteristics. The classification system, involving a list of parenting elements and variables, is presented in Table 2. The coded research characteristics employed in the final analyses can be found in Table 3. Based on the investigations included, the following data were entered into this MASEM: the correlations between constructs, the relevant sample sizes, year of publication, the mean age of the sample, individualism score for each country, the proportion of females, kinds of anti-social behavior, mode of assessment, self-control measurements, data extracted from which kind of design (cross-section vs. cross-section made through longitudinal research), the reliability of constructs. We used the definition and data of Hofstede to report the individualism score (https://www.hofstede-insights.com/country-comparison). We employed the correlation coefficient r to quantify the strength and direction of the links between constructs and also as the input of MASEM. Regarding the investigations that did not report the correlation of an aggregate measure between effective parenting practice, low self-control, and anti-social behaviors, an average correlation was calculated by computing the weighted mean of a list of correlations (more details are available on the Open Science Framework (https://osf.io/w9va6/)). We employed MASEM to explain the association between constructs and the indirect effect of effective parenting practice and their elements on anti-social behavior via low self-control. In this research, we used TSSEM to test the main hypotheses, and also both TSSEM and OSMASEM were applied to assess the effect of potential moderators.

One of the most comprehensive challenges facing researchers is how to apply and model meaningful effect size heterogeneity detected in the bivariate meta-analysis into Meta-Analytic Structural Equation Modeling (MA). The heterogeneity of effect size (i.e., a correlation coefficient between two variables) refers to the variability of estimates within a population (Higgins, [10]). effect size heterogeneity is essential because findings derived only from the analysis of pooled effect sizes are sometimes misleading and limited. Unfortunately, conventional MA approaches in applied social psychology [8] fail to explain the heterogeneity of effect size regarding the path coefficients of the model. Yu et al. ([11]) and Cheung [1] dealt with this problem and developed a set of techniques showing the variability surrounding relations in estimating model parameters (i.e., the heterogeneity of effect size) to calibrate the stability of parameters estimates across the population. We followed Yu et al.,’s ([11]) and Cheung's (2018) [1] combined guidelines to ensure the generalizability of findings (more details concerning the generalizability of estimated path coefficients are found in (https://osf.io/w9va6/)). The heterogeneity (SD) of the estimated parameter of the TSSEM was calibrated using the bootstrap method. In this method, random correlation matrices were sampled from the TSSEM-Stage one by the parametric bootstrap. The bootstrap method was based on the discussion in Cheung [1] and Yu et al., ([11]). Accordingly, when I^2^ and large-width CVs for each path coefficient values reveal the existence of heterogeneity, inconsistencies were explained by categorical moderator analyses through TSSEM and One Stage Meta-Analytic Structural Equation Modeling (OSMASEM), and also continuous moderator analyses through OSMASEM.

## 3 Sensitivity analyses and publication bias

**Fig. 1:**
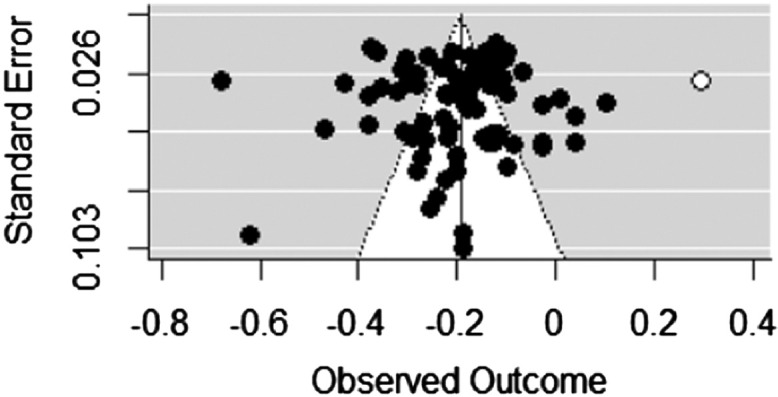
Effective parenting practices and low self-control.

**Fig. 2:**
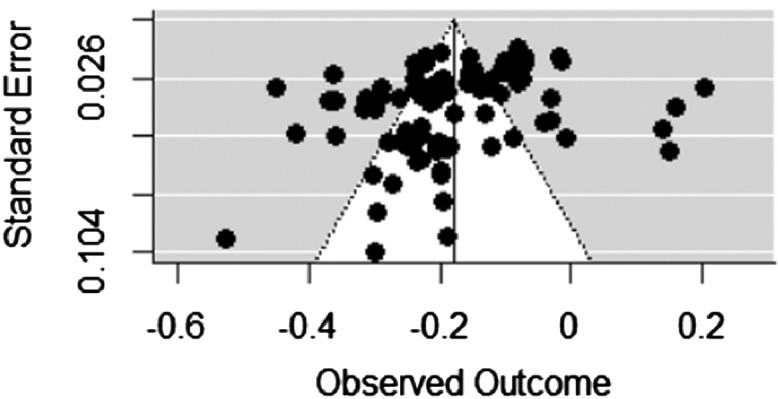
Effective parenting practices and Anti-social behavior.

**Fig. 3:**
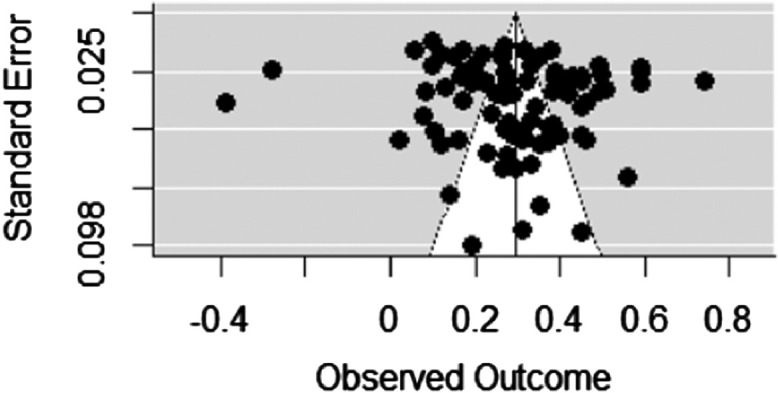
Low self-control and Anti-social behavior.

We performed outlier analyses to test the robustness of aggregated correlations among constructs. It was, however, performed on single correlations rather than correlation matrices. We applied SDRs to assess outlier analysis and also conducted different methods (i.e., TSSEM with corrected correlation, Univariate-r MASEM, FIMASEM, OSMASEM) to perform MASEM to check the robustness of the findings resulted from the principal method used in this meta-analysis (i.e., TSSEM). We also performed TSSEM on corrected correlation matrices, yet compared them for path coefficients with and without these corrections to test the sensitivity of our findings to measurement errors (unreliability corrections). Among the 85 samples included in the MASEM analysis, 11 samples did not report reliability for effective parenting, 8 for self-control, 13 for anti-social behaviors. We used means α 0.75, 0.77, and 0.77 for them, respectively. For better comparison between TSSEM with and without corrected correlation, all of the procedures are similar except that corrected correlations were pooled to generate correlation matrix. We assessed the robustness of the findings by comparing the findings of TSSEM with other approaches through which MASEM can be conducted (i.e., univariate-r MASEM, FIMASEM, and OSMASEM) (more details are found in (https://osf.io/w9va6/)). The current research adds multiple methods of assessing for publication bias, namely, the funnel plot, trim and fill method [5], file drawer analysis (Rosenthal, 1979), and Egger's linear regression test [6] to identify the robustness of findings and probable small research effect for single correlations (more details are found in (https://osf.io/w9va6/)). We used the R packages metaSEM to perform MASEM [2], and the metafor package to assess publication bias, outlier, and influential analyses (Viechtbauer & Cheung, 2010) (See R-code). In this research, the target p-value was equal to 0.05. If the 95% Confidence Intervals (95%-CIs) included zero, we concluded that the intended effect size is non-significant. Correlation based effect sizes were interpreted as small for r 〈 0.23, medium for *r* = 0.24 to 0.36, and large for r 〉 0.37) (Cohen, 1992) (See Fig. 1, Fig. 2, Fig. 3).

## Declaration of Competing Interest

The authors declare that they have no known competing financial interests or personal relationships that could have appeared to influence the work reported in this paper.

## Appendix Supplementary materials

Image, application 1
