# Supplementary material for: Dataset of parenting practices, self-control and anti-social behaviors: Meta-analytic structural equation modeling
Source: Data Brief. 2020 Aug 4;32:106114. doi: 10.1016/j.dib.2020.106114 (PMC7452709; doi:10.1016/j.dib.2020.106114)
Supplement: Supplementary file 1 [file mmc1.docx]

Parenting Practices, Self-Control and Anti-Social Behaviors: Meta-Analytic Structural Equation Modeling

**Overview**

| A | Literature review on fully or partially role of low self-control between parenting practices and anti-social behaviors | 2 |
| --- | --- | --- |
| B | List of Parenting Practices | 3 |
| C | Coded research characteristics used in analysis | 4 |
| D | Characteristics of included research | 5 |
| E | Funnel plot | 12 |
| F | The weighted mean of correlations | 13 |
| G | The generalizability of estimated path coefficients | 13 |
| H | Sensitivity analyses: checking the robustness of the results | 17 |
| I | Publication bias | 18 |
| J | References to studies used in this meta-analysis | 20 |
| K | Additional references | 28 |

Abbrevations:

| GTC: The General Theory of Crime | SEM: Structural Equation Modeling |
| --- | --- |
| MASEM: Meta-Analytic Structural Equation Modeling | TSSEM: Two-Stage meta-analytic Structural Equation Modeling |
| OSMASEM: One Stage Meta-Analytic Structural Equation Modeling | FIMASEM: Full Information Meta-Analytic Structural Equation Modeling |
| SDR_S_: studentized deleted residuals | WLS: Weighted Least Squares |

**Supplementary Material A: Literature review on fully or partially role of low self-control between parenting practices and anti-social behaviors**

Table supplementary A. Literature review on fully or partially role of low self-control between parenting practices and anti-social behaviors

|  | Researchers | Elements of parenting practices | Kinds of anti-social behavior |
| --- | --- | --- | --- |
| Full mediation model | Jo & Zhang (2014) | AEPP | AASB |
|  | Feldman & A. Weinberger (1994) | AEPP | AASB |
|  | Gibbs et al., (1998) | AEPP | AASB |
|  | Boisvert et al., (2012) | Attachment | AASB |
|  | Cochran et al., (1998) | AEPP | Academic dishonesty |
|  | E. Higgins (2002) | AEPP | AASB |
|  | Simons el al (2007) | Monitoring/  Discipline | AASB |
|  |  | Supportive involvement |  |
|  |  | Hostility/  Rejection |  |
| Partial mediation model | Muftić et al., (2014) | AEPP | Violence perpetration |
|  |  | AEPP | Property offending |
|  | C. Lagrange (1999) | Supervision | Violent offenses |
|  | Hay (2001) | Monitoring/  Discipline | AASB |
|  | Gibbs et al., (2003) | AEPP | AASB |
|  | Vazsonyi & Belliston (2007) | Support | AASB |
|  |  | Monitoring | AASB |
|  | Benda (2003) | AEPP | AASB |
|  | Chapple et al., (2005) | Monitoring | Substance Use |
|  | Finkenauer et al., (2005) | AEPP | Behavioral problems |
|  | Jones et al.,(2007) | Support | AASB |
|  | Kort-Butler et al., (2011) | Monitoring | Criminal behavior |
|  | Morris et al., (2007) | AEPP | AASB |
|  | Perrone et al., (2004) | AEPP | AASB |
|  | Unnever et al.,(2003) | AEPP | AASB |
|  | Boisvert et al., (2012) | Rejection | AASB |
| *Note*. AEPP is Aggregated construct of Effective Parenting Practices; AASB is Aggregated construct of Anti-Social Behavior. | | | |

**Supplementary Material B. List of Parenting Practices**

Table supplementary B. List of Parenting Practices

| The element of parenting practices | Parenting behaviors | Names and words in description |
| --- | --- | --- |
| Emotionally supportive practices | | |
|  | Affection | Warmth |
|  |  | Affection |
|  |  | Acceptance |
|  |  | Affective tie |
|  |  | Hugs |
|  |  | Loving |
|  |  | Positive feelings |
|  |  | Smiles |
|  |  | Intimate relationship |
|  | Support | Emotional support |
|  |  | Understanding |
|  |  | Helpful |
|  |  | Encouraging |
|  |  | Trust |
|  | Closeness | Involvement |
|  |  | Cohesion |
|  |  | Attachment |
|  |  | Attention |
|  |  | Pay attention |
|  |  | Care |
|  | Neglectful (−) | Neglect |
|  |  | Avoidance |
|  | Rejection (−) | Rejection |
|  |  | Conflict |
|  |  | Withdrawal |
|  | Hostility (−) | Hostility |
|  |  | Anger |
|  |  | Annoyance |
|  |  | Irritation |
|  |  | Sarcasm |
| Monitoring | | |
|  | Supervision | Tracking of activities |
|  |  | Tracking of whereabouts |
|  |  | Tracking the child's behavior |
|  |  | Checking homework |
|  |  | Awareness of activities |
| Recognition | | |
|  | Recognize the anti-social behaviors | The ability of the parent to recognize when youth engage in anti-social behaviors |
| Effective discipline | | |
|  | Fair and Non-corporal means of punishment | Calmly discuss misbehavior |
|  |  | Noticing when doing good |
|  |  | Withdrawal of privileges |
|  |  | Consistent discipline |
|  |  | Proportionate punishment |
|  |  | Agree on discipline |
|  |  | Responsive discipline |
|  | Harsh discipline(−) | Firm control |
|  |  | Harsh punishment |
|  | Physical punishment(−) | Beaten child up |
|  |  | Hitting |
|  |  | Kicking |
|  |  | Slapping |
|  | Verbal aggression as punishment(−) | Abusive name calling |
|  |  | Yelling |
|  |  | Nagging |
|  |  | Scolding |
|  |  | Verbal attacks |
|  |  | Threatening to hit |
| *Note.* This study equalized the direction of effect sizes (multiplied by −1 as needed), for neglectful, rejection, hostility, harsh discipline, verbal aggression, and physical punishment as the different manifestations of parenting practices’ elements, to display relationships of effective parenting practices with low self-control and anti-social behaviors. | | |

**Supplementary Material C. Coded study characteristics used in analysis**

Table supplementary C. Coded study characteristics used in analysis.

| **Characteristic** | **Coded as** | **Used to** |
| --- | --- | --- |
| Sample size (n) | (C) Number of participants included in the analysis | Weight each study findings |
| Sex (female) | (C) Proportion of female respondents | Proportion of female, mean age and culture used as a continues moderators on the relationship of effective parenting practices,low self-control, and anti-social behavior. |
| Age | (C) Mean age of the sample |  |
| Culture | (C) Hofstede’s individualism score |  |
| Mode of assessment | (CA)The perspective from which participants' low self-control and anti-social behavioars were assessed. Due to a small number of alternative categories coded as self-report (1) vs other (0). |  |
| Type of anti-social behaviors | (CA) vandalism, theft, and assault, group fight, shot or stabbed someone, and pulled a knife or a gun on someone , physical assault, shoplifting, carry a hidden weapon, attack someone categories coded as crime (1)  Alcohol use, school misconduct, sell drugs, write bad checks, gang membership, nonviolent crime, substances use, childhood antisociality, risky lifestyles, running away home, risk-taking behaviors categories coded as analogous behavior (2), and general deviance (3). |  |
| Data extracted from which kinds of design | (CA) Data extracted from cross-sectional studies versus cross-section made through longitudinal studies. Categories coded as cross-section (1) and cross-section made through longitudinal studies (2). |  |
| Self-control measurements | (CA) Due to a small number of alternative categories coded as Grasmick (1993) constructed a 24-item (1) and other measurements (2). |  |
| *Note*. (C) = continuous, (CA) = categorical variables. | | |

**Supplementary Material D. Characteristics of included studies**

Table supplementary D. Characteristics of included studies

| Author name (Year) (Country) | Sample Size | Female % | Mean age | IND  score | Kinds  ASB | MODEASS | LSC MEAS | Kinds of design | Alpha Parenting | Alpha LSC | Alpha ASB |
| --- | --- | --- | --- | --- | --- | --- | --- | --- | --- | --- | --- |
| (Vazsonyi et al., 2016) (Czech) | | | | | | | | | | | |
| Setting 1 | 239 | 47.5 | 14.02 | 58 | AB | SR | GR93 | CS | .71 | .83 | .88 |
| Setting 2 | 239 | 47.5 | 14.02 | 58 | AB | SR | GR93 | CS | .71 | .83 | .67 |
| Setting 3 | 239 | 47.5 | 14.02 | 58 | CR | SR | GR93 | CS | .71 | .83 | .76 |
| Setting 4 | 130 | 47.7 | 14.71 | 58 | AB | SR | GR93 | CS | .79 | .82 | .88 |
| Setting 5 | 130 | 47.7 | 14.71 | 58 | AB | SR | GR93 | CS | .79 | .82 | .70 |
| Setting 6 | 130 | 47.7 | 14.71 | 58 | CR | SR | GR93 | CS | .79 | .82 | .87 |
| (Alvarez-Rivera & Fox, 2010) (Puerto Rico) | 298 | 54 | 16.23 | 27 | AB | SR | GR93 | CS | .92 | .68 | .82 |
| (Anderson et al., 2015) (U.S.) | | | | | | | | | | | |
| Setting 1 | 1072 | 51.4 | 13.53 | 91 | AB | SR | OTHER | LON | .72 | .65 | .94 |
| Setting 2 | 1072 | 51.4 | 13.53 | 91 | CR | SR | OTHER | LON | .72 | .65 | .86 |
| Setting 3 | 1072 | 51.4 | 13.53 | 91 | CR | SR | OTHER | LON | .72 | .65 |  |
| (Baker, 2010) (U.S.) | 4834 | 52 | 16.02 | 91 | AB | OTHER | OTHER | LON | .78 | .68 |  |
| (Bobbio et al., 2019) (Argentina) | 214 | 00 | 15.89 | 46 | CR | SR | GR93 | CS | .8 | .85 | .76 |
| (Boccio & Beaver, 2018)(U.S.) | | | | | | | | | | | |
| Setting 1 | 346 | 50 | 15.5 | 91 | CR | OTHER | OTHER | LON | .55 | .66 | .6 |
| Setting 2 | 346 | 50 | 15.5 | 91 | CR | OTHER | OTHER | LON | .64 | .66 | .6 |
| Setting 3 | 346 | 50 | 15.5 | 91 | CR | OTHER | OTHER | LON | .84 | .66 | .6 |
| Setting 4 | 346 | 50 | 15.5 | 91 | AB | OTHER | OTHER | LON | .55 | .66 | .53 |
| Setting 5 | 346 | 50 | 15.5 | 91 | AB | OTHER | OTHER | LON | .64 | .66 | .53 |
| Setting 6 | 346 | 50 | 15.5 | 91 | AB | OTHER | OTHER | LON | .84 | .66 | .53 |
| (Brownfield, 2010) (Canada) | 618 | 52.8 | 14 | 80 | AB | SR | OTHER | CS |  |  |  |
| (Burt & Ronald, 2006) (U.S.) | 754 | 53 | 13 | 91 | CR | OTHER | GR93 | LON | .77 | .89 | .9 |
| (Cheung & Cheung, 2008) (Hong Kong) | | | | | | | | | | | |
| Setting 1 | 1015 | 54 | 16.01 | 25 | AB | SR | OTHER | CS | .66 | .61 | .73 |
| Setting 2 | 1015 | 54 | 16.01 | 25 | CR | SR | OTHER | CS | .66 | .61 | .5 |
| Setting 3 | 1015 | 54 | 16.01 | 25 | CR | SR | OTHER | CS | .66 | .61 | .5 |
| Setting 4 | 1015 | 54 | 16.01 | 25 | CR | SR | OTHER | CS | .56 | .61 | .73 |
| Setting 5 | 1015 | 54 | 16.01 | 25 | CR | SR | OTHER | CS | .56 | .61 | .5 |
| Setting 6 | 1015 | 54 | 16.01 | 25 | CR | SR | OTHER | CS | .56 | .61 | .5 |
| (Cheung & Cheung, 2010) (Hong Kong) | | | | | | | | | | | |
| Setting 1 | 1015 | 54 | 16 | 25 | CR | SR | OTHER | CS | .66 | .61 | .79 |
| Setting 2 | 1015 | 54 | 16 | 25 | CR | SR | OTHER | CS | .56 | .61 | .7 |
| (Costello & Dunaway, 2003) (U.S.) | | | | | | | | | | | |
| Setting 1 | 377 | 52.51 | 15 | 91 | CR | SR | GR93 | CS | .84 | .78 | .64 |
| Setting 2 | 377 | 52.51 | 15 | 91 | AB | SR | GR93 | CS | .84 | .78 | .84 |
| (Evans et al., 2012) (U.S.) | | | | | | | | | | | |
| Setting 1 | 381 | 100 | 13 | 91 | GC | SR | OTHER | LON | .77 | .73 | .9 |
| Setting 2 | 381 | 100 | 13 | 91 | GC | SR | OTHER | LON | .70 | .73 | .9 |
| Setting 3 | 381 | 100 | 13 | 91 | GC | SR | OTHER | LON | .88 | .73 | .9 |
| Setting 4 | 381 | 100 | 13 | 91 | GC | SR | OTHER | LON | .71 | .73 | .9 |
| Setting 5 | 381 | 0 | 13 | 91 | GC | SR | OTHER | LON | .77 | .73 | .9 |
| Setting 6 | 381 | 0 | 13 | 91 | GC | SR | OTHER | LON | .70 | .73 | .9 |
| Setting 7 | 381 | 0 | 13 | 91 | GC | SR | OTHER | LON | .88 | .73 | .9 |
| Setting 8 | 381 | 0 | 13 | 91 | GC | SR | OTHER | LON | .71 | .73 | .9 |
| (Frijns et al.,2005)  (Netherlands) | 1173 | 49 | 12.3 | 80 | GC | SR | OTHER | LON | .79 | .7 | .93 |
| (Guo, 2018)(U.S.) | 1020 | 50 | 12.23 | 91 | GC | SR | OTHER | CS | .68 | .83 | .65 |
| (Hay, 2001) (U.S.) | | | | | | | | | | | |
| Setting 1 | 197 | 50 | 16 | 91 | AB | SR | GR93 | CS | .79 | .81 | .45 |
| Setting 2 | 197 | 50 | 16 | 91 | AB | SR | GR93 | CS | .45 | .81 | .45 |
| Setting 3 | 197 | 50 | 16 | 91 | AB | SR | GR93 | CS | .88 | .81 | .45 |
| Setting 4 | 197 | 50 | 16 | 91 | AB | SR | GR93 | CS | .77 | .81 | .45 |
| Setting 5 | 197 | 50 | 16 | 91 | AB | SR | GR93 | CS | .85 | .81 | .45 |
| Setting 6 | 197 | 50 | 16 | 91 | AB | SR | GR93 | CS | .79 | .81 | .61 |
| Setting 7 | 197 | 50 | 16 | 91 | AB | SR | GR93 | CS | .45 | .81 | .61 |
| Setting 8 | 197 | 50 | 16 | 91 | AB | SR | GR93 | CS | .88 | .81 | .61 |
| Setting 9 | 197 | 50 | 16 | 91 | AB | SR | GR93 | CS | .77 | .81 | .61 |
| Setting 10 | 197 | 50 | 16 | 91 | AB | SR | GR93 | CS | .85 | .81 | .61 |
| (Hay & Forrest, 2008) (U.S.) | 750 | 52 | 13.22 | 91 | GC | SR | OTHER | CS | .79 | .63 |  |
| (Higgins, 2002) (U.S.) | 425 | 52.9 | 21 | 91 | GC | SR | OTHER | CS | .92 | .91 | .8 |
| (Huang, 2007) (U.S.) | 985 | 50.6 | 11 | 91 | AB | OTHER | OTHER | LON | .7 | .8 | .8 |
| (Intravia et al., 2012) (U.S.) | 1675 | 50 | 13.79 | 91 | CR | SR | OTHER | CS |  |  | .84 |
| (Schreck et al., 2002) (U.S.) | 1101 | 51 | 15.5 | 91 | AB | SR | GR93 | CS |  |  | .84 |
| (Unnever et al., 2006) (U.S.) | | | | | | | | | | | |
| Setting 1 | 2472 | 51.1 | 12.39 | 91 | AB | SR | GR93 | CS | .77 | .87 | .78 |
| Setting 2 | 2472 | 51.1 | 12.39 | 91 | AB | SR | GR93 | CS | .74 | .87 | .78 |
| Setting 3 | 2472 | 51.1 | 12.39 | 91 | AB | SR | GR93 | CS |  | .87 | .78 |
| Setting 4 | 2472 | 51.1 | 12.39 | 91 | AB | SR | GR93 | CS | .72 | .87 | .78 |
| Setting 5 | 2472 | 51.1 | 12.39 | 91 | AB | SR | GR93 | CS | .77 | .87 | .82 |
| Setting 6 | 2472 | 51.1 | 12.39 | 91 | AB | SR | GR93 | CS | .74 | .87 | .82 |
| Setting 7 | 2472 | 51.1 | 12.39 | 91 | AB | SR | GR93 | CS |  | .87 | .82 |
| Setting 8 | 2472 | 51.1 | 12.39 | 91 | AB | SR | GR93 | CS | .72 | .87 | .8 |
| (Janssen et al., 2016)  (Netherlands) | 615 | 48 | 13.9 | 80 | GC | SR | GR93 | LON | .82 | .72 | .83 |
| (Janssen et al., 2017) (Netherlands) | | | | | | | | | | | |
| Setting 1 | 315 | 00 | 13 | 80 | GC | SR | GR93 | LON | .77 | .75 | .85 |
| Setting 2 | 288 | 100 | 13 | 80 | GC | SR | GR93 | LON | .77 | .75 | .85 |
| (Jennings et al., 2010) (U.S.) | 407 | 58.3 | 16 | 91 | AB | SR | OTHER | CS | .75 | .83 |  |
| (Jeon & Chun, 2017) (South Korea) | | | | | | | | | | | |
| Setting 1 | 3449 | 50 | 14 | 18 | AB | SR | OTHER | CS | .86 | .65 | .54 |
| Setting 2 | 3449 | 50 | 14 | 18 | AB | SR | OTHER | CS | .86 | .65 | .64 |
| (Youngoh Jo & Lee, 2018) (South Korea) | 2491 | 85.3 | 11 | 18 | CR | SR | GR93 | LON | .82 | .64 | .55 |
| (Junger & Tremblay, 1999) (Canada) | | | | | | | | | | | |
| Setting 1 | 731 | 00 | 13.5 | 80 | CR | SR | GR93 | LON | .81 | .85 | .92 |
| Setting 2 | 731 | 00 | 13.5 | 80 | CR | SR | GR93 | LON | .81 | .85 | .79 |
| (Kazemian et al., 2009) (Canada) | 470 | 00 | 16.9 | 80 | AB | OTHER | OTHER | LON | .68 | .75 | .62 |
| (Kuhn & Laird, 2013) (U.S.) | | | | | | | | | | | |
| Setting 1 | 180 | 51 | 12.04 | 91 | AB | SR | GR93 | CS | .79 | .88 | .87 |
| Setting 2 | 180 | 51 | 12.04 | 91 | AB | SR | GR93 | CS | .58 | .88 | .87 |
| (Li et al., 2019) (Poland) | | | | | | | | | | | |
| Setting 1 | 146 | 00 | 16.97 | 60 | AB | SR | OTHER | CS | .95 | .77 | .77 |
| Setting 2 | 146 | 00 | 16.97 | 60 | AB | SR | OTHER | CS | .91 | .77 | .77 |
| Setting 3 | 355 | 00 | 16.97 | 60 | AB | SR | OTHER | CS | .95 | .77 | .77 |
| Setting 4 | 355 | 00 | 16.97 | 60 | AB | SR | OTHER | CS | .91 | .77 | .77 |
| (Longshore et al., 2005) (U.S.) | 359 | 26 | 16 | 91 | CR | SR | OTHER | LON | .51 | .54 | .58 |
| (Kort-Butler et al., 2011) (U.S.) | | | | | | | | | | | |
| Setting 1 | 199 | 40 | 21.5 | 91 | CR | OTHER | GR93 | CS | .75 | .82 | .89 |
| Setting 2 | 199 | 40 | 21.5 | 91 | CR | OTHER | GR93 | CS | .88 | .82 | .89 |
| Setting 3 | 199 | 40 | 21.5 | 91 | CR | OTHER | GR93 | CS | .83 | .82 | .89 |
| Setting 4 | 199 | 40 | 21.5 | 91 | CR | OTHER | GR93 | CS | .75 | .82 | .78 |
| Setting 5 | 199 | 40 | 21.5 | 91 | CR | OTHER | GR93 | CS | .88 | .82 | .78 |
| Setting 6 | 199 | 40 | 21.5 | 91 | CR | OTHER | GR93 | CS | .83 | .82 | .78 |
| (McGloin et al., 2004) (U.S.) | 1725 | 49 | 12.73 | 91 | AB | OTHER | GR93 | LON |  | .78 | .85 |
| (McKee, 2012) (U.S.) | 1409 | 54 | 12.2 | 91 | GC | SR | GR93 | LON | .72 | .7 | .85 |
| (Meldrum, 2008) (U.S.) | 1034 | 48 | 12.07 | 91 | CR | OTHER | OTHER | LON |  | .64 |  |
| (Meldrum et al., 2013) (U.S.) | 825 | 50 | 15 | 91 | GC | SR | OTHER | LON | .91 | .82 | .82 |
| (Meldrum et al., 2015) (U.S.) | | | | | | | | | | | |
| Setting 1 | 101 | 22 | 15.67 | 91 | AB | OTHER | GR93 | CS | .85 | .92 |  |
| Setting 2 | 101 | 22 | 15.67 | 91 | AB | OTHER | GR93 | CS | .89 | .92 |  |
| Setting 3 | 101 | 22 | 15.67 | 91 | AB | OTHER | GR93 | CS | .88 | .92 |  |
| (Meldrum, et al., 2009) (U.S.) | 1364 | 46 | 14.03 | 91 | AB | SR | GR93 | LON | .8 | .78 | .61 |
| (Miller, 2012) (U.S.) | | | | | | | | | | | |
| Setting 1 | 763 | 49 | 11.93 | 91 | AB | SR | OTHER | LON | .69 | .68 |  |
| Setting 2 | 763 | 49 | 11.93 | 91 | CR | SR | OTHER | LON | .69 | .68 |  |
| (Moon & Morash, 2013) (U.S.) | | | | | | | | | | | |
| Setting 1 | 296 | 57 | 14 | 91 | CR | SR | GR93 | CS | .65 | .9 | .91 |
| Setting 2 | 296 | 57 | 14 | 91 | CR | SR | GR93 | CS | .84 | .9 | .91 |
| Setting 3 | 296 | 57 | 14 | 91 | CR | SR | GR93 | CS | .85 | .9 | .91 |
| Setting 4 | 296 | 57 | 14 | 91 | GC | SR | GR93 | CS | .65 | .9 | .79 |
| Setting 5 | 296 | 57 | 14 | 91 | GC | SR | GR93 | CS | .85 | .9 | .79 |
| Setting 6 | 296 | 57 | 14 | 91 | GC | SR | GR93 | CS | .85 | .9 | .79 |
| Setting 7 | 296 | 57 | 14 | 91 | CR | SR | GR93 | CS | .65 | .9 | .82 |
| Setting 8 | 296 | 57 | 14 | 91 | GC | SR | GR93 | CS | .84 | .9 | .82 |
| Setting 9 | 296 | 57 | 14 | 91 | GC | SR | GR93 | CS | .85 | .9 | .79 |
| (Moon & Alarid, 2015) (U.S.) | 296 | 57 | 14 | 91 | GC | SR | GR93 | CS | .9 | .9 | .88 |
| (Muftić et al., 2014) (U.S.) | 1759 | 50.50 | 13.79 | 91 | GC | SR | GR93 | LON |  | .85 | .73 |
| (Kabiri et al., 2019) (Iran) | 784 | 44 | 24.3 | 41 | AB | SR | GR93 | CS | .88 | .88 | .89 |
| (Schreck, 2002) (U.S.) | 1054 | 51 | 16 | 91 | CR | SR | GR93 | CS |  |  |  |
| (Simons et al., 2007) (U.S.) | | | | | | | | | | | |
| Setting 1 | 867 | 54 | 10.5 | 91 | AB | SR | OTHER | LON | .75 | .8 |  |
| Setting 2 | 867 | 54 | 10.5 | 91 | AB | SR | OTHER | LON | .83 | .8 |  |
| Setting 3 | 867 | 54 | 10.5 | 91 | AB | SR | OTHER | LON | .79 | .8 |  |
| (Vazsonyi et al., 2007) (Hungry) | | | | | | | | | | | |
| Setting 1 | 826 | 31.6 | 16.6 | 80 | GC | SR | GR93 | CS | .79 | .83 | .96 |
| Setting 2 | 826 | 31.6 | 16.6 | 80 | GC | SR | GR93 | CS | .70 | .83 | .96 |
| Setting 3 | 826 | 31.6 | 16.6 | 80 | GC | SR | GR93 | CS | .75 | .83 | .96 |
| (Vazsonyi & Belliston, 2007) (Japan) | | | | | | | | | | | |
| Setting 1 | 344 | 66.6 | 19.8 | 46 | GC | SR | GR93 | CS | .75 | .8 | .91 |
| Setting 2 | 344 | 66.6 | 19.8 | 46 | GC | SR | GR93 | CS | .74 | .8 | .91 |
| Setting 3 | 344 | 66.6 | 19.8 | 46 | GC | SR | GR93 | CS | .69 | .8 | .91 |
| (Vazsonyi & Belliston, 2007) ( Netherland) | | | | | | | | | | | |
| Setting 1 | 1244 | 53.3 | 16.10 | 80 | GC | SR | GR93 | CS | .74 | .85 | .95 |
| Setting 2 | 1244 | 53.3 | 16.10 | 80 | GC | SR | GR93 | CS | .73 | .85 | .95 |
| Setting 3 | 1244 | 53.3 | 16.10 | 80 | GC | SR | GR93 | CS | .72 | .85 | .95 |
| (Vazsonyi & Belliston, 2007) ( Switzerland) | | | | | | | | | | | |
| Setting 1 | 3819 | 37.5 | 18.2 | 68 | GC | SR | GR93 | CS | .74 | .8 | .96 |
| Setting 2 | 3819 | 37.5 | 18.2 | 68 | GC | SR | GR93 | CS | .75 | .8 | .96 |
| Setting 3 | 3819 | 37.5 | 18.2 | 68 | GC | SR | GR93 | CS | .79 | .8 | .96 |
| (Vazsonyi & Belliston, 2007) (U.S.) | | | | | | | | | | | |
| Setting 1 | 1273 | 61.4 | 20 | 91 | GC | SR | GR93 | CS | .87 | .85 | .95 |
| Setting 2 | 1273 | 61.4 | 20 | 91 | GC | SR | GR93 | CS | .83 | .85 | .95 |
| Setting 3 | 1273 | 61.4 | 20 | 91 | GC | SR | GR93 | CS | .79 | .85 | .95 |
| (Vazsonyi & Belliston, 2007) (U.S.) | | | | | | | | | | | |
| Setting 1 | 802 | 49.9 | 16.4 | 91 | GC | SR | GR93 | CS | .84 | .91 | .97 |
| Setting 2 | 802 | 49.9 | 16.4 | 91 | GC | SR | GR93 | CS | .83 | .91 | .97 |
| Setting 3 | 802 | 49.9 | 16.4 | 91 | GC | SR | GR93 | CS | .78 | .91 | .97 |
| (Vazsonyi & Belliston, 2007) ( U.S.) | | | | | | | | | | | |
| Setting 1 | 689 | 53.6 | 15.7 | 91 | GC | SR | GR93 | CS | .88 | .92 | .99 |
| Setting 2 | 689 | 53.6 | 15.7 | 91 | GC | SR | GR93 | CS | .83 | .92 | .99 |
| Setting 3 | 689 | 53.6 | 15.7 | 91 | GC | SR | GR93 | CS | .85 | .92 | .99 |
| (Vazsonyi & Klanjšek, 2008) (Switzerland) | | | | | | | | | | | |
| Setting 1 | 2603 | 29.5 | 18.22 | 68 | GC | SR | GR93 | CS | .74 | .8 | .96 |
| Setting 2 | 2603 | 29.5 | 18.22 | 68 | GC | SR | GR93 | CS | .76 | .8 | .96 |
| Setting 3 | 2603 | 29.5 | 18.22 | 68 | GC | SR | GR93 | CS | .78 | .8 | .96 |
| Setting 4 | 2603 | 29.5 | 18.22 | 68 | GC | SR | GR93 | CS | .81 | .8 | .96 |
| Setting 5 | 2603 | 29.5 | 18.22 | 68 | GC | SR | GR93 | CS | .76 | .8 | .96 |
| Setting 6 | 2603 | 29.5 | 18.22 | 68 | GC | SR | GR93 | CS | .85 | .8 | .96 |
| Setting 7 | 2603 | 29.5 | 18.22 | 68 | GC | SR | GR93 | CS | .73 | .94 | .94 |
| Setting 8 | 2603 | 29.5 | 18.22 | 68 | GC | SR | GR93 | CS | .71 | .94 | .94 |
| Setting 9 | 2603 | 29.5 | 18.22 | 68 | GC | SR | GR93 | CS | .8 | .94 | .94 |
| Setting 10 | 2603 | 29.5 | 18.22 | 68 | GC | SR | GR93 | CS | .77 | .94 | .94 |
| Setting 11 | 2603 | 29.5 | 18.22 | 68 | GC | SR | GR93 | CS | .86 | .94 | .94 |
| (Vazsonyi & Huang, 2010) (U.S.) | 1364 | 48.8 | 10.5 | 91 | GC | SR | OTHER | LON | .73 | .81 | .8 |
| (Vera & Moon, 2013) (U.S.) | | | | | | | | | | | |
| Setting 1 | 277 | 57 | 14 | 91 | AB | SR | GR93 | CS | .84 | .9 | .94 |
| Setting 2 | 277 | 57 | 14 | 91 | AB | SR | GR93 | CS | .84 | .9 | .94 |
| (Jr et al., 1998) (U.S.) | 555 | 50 | 40.5 | 91 | GC | SR | GR93 | CS | .86 | .64 | .6 |
| (Wright et al., 2001) (New Zealand) | 1037 | 49 | 16 | 79 | GC | SR | OTHER | CS |  |  |  |
| (You & Kim, 2016) (South Korea) | | | | | | | | | | | |
| Setting 1 | 448 | 00 | 15.2 | 18 | AB | SR | OTHER | CS | .94 | .75 | .84 |
| Setting 2 | 282 | 100 | 15.2 | 18 | AB | SR | OTHER | CS | .94 | .75 | .84 |
| (Beaver, 2008) (Canada) | | | | | | | | | | | |
| Setting 1 | 3780 | 50 | 8 | 80 | CR | SR | OTHER | LON | .72 | .79 | .52 |
| Setting 2 | 3780 | 50 | 8 | 80 | CR | SR | OTHER | LON | .66 | .79 | .52 |
| Setting 3 | 3780 | 50 | 8 | 80 | CR | SR | OTHER | LON | .57 | .79 | .52 |
| (Chen, 2017)(China) | 600 | 50 | 8 | 20 | AB | SR | GR93 | CS | .71 |  | .86 |
| (Cho et al., 2005) (South Korea) | 2844 | 46 | 8 | 18 | AB | SR | 2 OTHER | LON | .83 | .8 | .75 |
| (Finkenauer et al., 2005) (Netherland) | | | | | | | | | | | |
| Setting 1 | 1359 | 47.8 | 12.3 | 80 | CR | SR | OTHER | CS | .8 | .67 | .84 |
| Setting 2 | 1359 | 47.8 | 12.3 | 80 | CR | SR | OTHER | CS | .65 | .67 | .84 |
| Setting 3 | 1359 | 47.8 | 12.3 | 80 | CR | SR | OTHER | CS | .68 | .67 | .84 |
| Setting 4 | 1359 | 47.8 | 12.3 | 80 | CR | SR | OTHER | CS | .8 | .67 | .68 |
| Setting 5 | 1359 | 47.8 | 12.3 | 80 | CR | SR | OTHER | CS | .65 | .67 | .68 |
| Setting 6 | 1359 | 47.8 | 12.3 | 80 | CR | SR | OTHER | CS | .68 | .67 | .68 |
| (Liu et al., 2019) (china) | | | | | | | | | | | |
| Setting 1 | 917 | 46.23 | 14.38 | 20 | AB | SR | OTHER | CS | .94 | .88 | .71 |
| Setting 2 | 917 | 46.23 | 14.38 | 20 | AB | SR | OTHER | CS | .9 | .88 | .71 |
| (Marcotte et al., 2002) (Canada) | | | | | | | | | | | |
| Setting 1 | 249 | 00 | 15.09 | 80 | AB | SR | OTHER | CS | .94 | .63 | .71 |
| Setting 2 | 279 | 100 | 15.09 | 80 | AB | SR | OTHER | CS | .94 | .63 | .71 |
| (Moon et al., 2012) (South Korea) | | | | | | | | | | | |
| Setting 1 | 2817 | 49 | 14 | 18 | AB | SR | GR93 | LON | .87 | .63 | .67 |
| Setting 2 | 2817 | 49 | 14 | 18 | AB | SR | GR93 | LON | .82 | .63 | .67 |
| (Özdemir et al., 2013) (Turkey) | | | | | | | | | | | |
| Setting 1 | 546 | 56.2 | 16 | 37 | AB | SR | GR93 | CS | .77 | .83 | .8 |
| Setting 2 | 546 | 56.2 | 16 | 37 | AB | SR | GR93 | CS | .86 | .83 | .8 |
| (Shadmanfaat et al., 2018) (Iran) | 318 | 48 | 23 | 41 | AB | SR | GR93 | CS |  |  |  |
| (Cho et al., 2019) (South Korea) | 2844 | 50 | 13 | 18 | GC | SR | OTHER | CS | .86 | .8 |  |
| (Watts & McNulty, 2016)(U.S.) | 3610 | 00 | 16.11 | 91 | GC | OTHER | OTHER | LON | .81 | .71 | .74 |
| (Wells et al., 2015) (U.S.) | 102 | 00 | 31.73 | 91 | CR | SR | GR93 | CS | .86 | .9 | .94 |
| (Woeckener et al., 2018) (U.S.) | 322 | 69.25 | 19.27 | 91 | GC | SR | GR93 | CS | .8 | .84 | .79 |
| (Gelder et al., 2017) (Switzerland) | 1197 | 48 | 15.04 | 68 | AB | SR | GR93 | LON |  |  |  |
| (Chae, 2016) (U.S.) | 6504 | 51.61 | 9.5 | 91 | AB | OTHER | GR93 | LON | .84 | .69 | .73 |
| (Toro, 2010) (U.S.) | | | | | | | | | | | |
| Setting 1 | 89 | 61.79 | 15.58 | 91 | AB | SR | 1 | CS | .68 | .85 |  |
| Setting 2 | 89 | 61.79 | 15.58 | 91 | AB | SR | 1 | CS | .7 | .85 |  |
| Setting 3 | 89 | 61.79 | 15.58 | 91 | AB | SR | 1 | CS | .78 | .85 |  |
| (Morris, 2003) (U.S.) | 1504 | 58.4 | 17 | 91 | GC | SR | 1 | CS | .64 | .87 | .88 |
| (Owens-Sabir, 2005) (U.S.) | | | | | | | | | | | |
| Setting 1 | 1724 | 56.6 | 17.2 | 91 | CR | SR | 1 | CS | .64 | .88 | .76 |
| Setting 2 | 1724 | 56.6 | 17.2 | 91 | CR | SR | 1 | CS | .64 | .88 | .61 |
| Setting 3 | 1724 | 56.6 | 17.2 | 91 | CR | SR | 1 | CS | .64 | .88 | .8 |
| Setting 4 | 1724 | 56.6 | 17.2 | 91 | CR | SR | 1 | CS | .64 | .88 | .74 |
| Setting 5 | 1724 | 56.6 | 17.2 | 91 | CR | SR | 1 | CS | .72 | .88 | .76 |
| Setting 6 | 1724 | 56.6 | 17.2 | 91 | CR | SR | 1 | CS | .72 | .88 | .61 |
| Setting 7 | 1724 | 56.6 | 17.2 | 91 | CR | SR | 1 | CS | .72 | .88 | .8 |
| Setting 8 | 1724 | 56.6 | 17.2 | 91 | CR | SR | 1 | CS | .72 | .88 | .74 |
| (Brauer, 2011) (U.S.) | 1919 | 50 | 13.5 | 91 | GC | SR | 2 | LON |  | .6 | .72 |
| *Note.* IND score=individualism score; Kinds ASB= kinds of anti-social behavior; MODE ASS= mode of assessment; LSC MEAS= Low Self-Control measurement; Alpha_ASB= alpha anti-social behavior Analogous Behavior=AB; Crime= CR; General Crime= GC; Self Report =SR; Cross Section=CS; Longitudinal = LON; Grasmick(1993)= GR93 | | | | | | | | | | | |

**Supplementary Material E. Funnel plot**

**Figure1**

Effective parenting practices and Low self-control


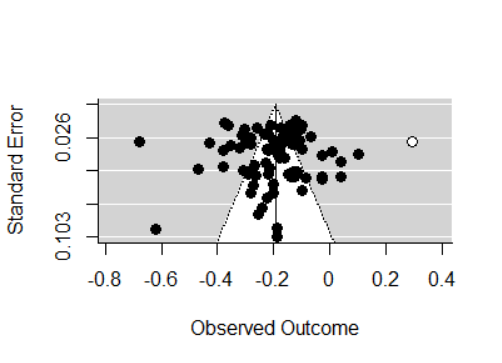


**Figure2**

Effective parenting practices and Anti-social behavior


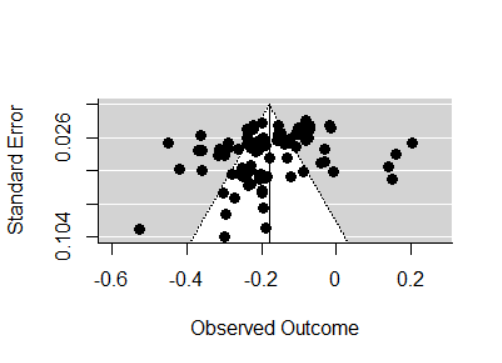


**Figure3**

Low self-control and Anti-social behavior


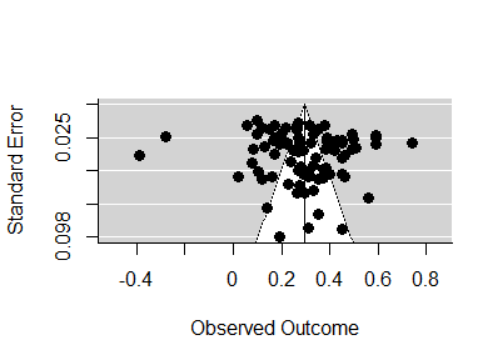


**Supplementary Material F. The weighted mean of correlations**

On averaging correlations, the usual approach is either to average the observed correlations or to average the Fisher's z transformed rs and to back-transform the average z value. However, there is an alternative estimator that is superior to these procedures (Alexander, 1990). Olkin and Pratt (1958) introduced an approximately unbiased minimum-variance estimator, which was better than other methods and was also simple to compute. Eid et al. (2010) recommend applying the correction of Olkin and Pratt (1958) instead of traditional Fisher z approach, as simulations proved it to estimate the average correlation more accurately. The algorithm of Olkin and Pratt (1958) was computed according to the psychometrica calculator (<https://www.psychometrica.de/correlation.html>).

**Supplementary Material G. The generalizability of estimated path coefficients**

The heterogeneity of effect size refers to the variability of estimates within a population (Higgins, 2008). Incorporating effect size heterogeneity is essential because results derived only from the analysis of pooled effect sizes are sometimes misleading and limited. One of the most comprehensive challenges facing researchers is how to apply and model meaningful effect size heterogeneity detected in the bivariate meta-analysis into Meta-Analytic Structural Equation Modeling (MA). Unfortunately, conventional MA approaches in applied social psychology ( i.e., Viswesvaran & Ones, 1995) fail to explain the heterogeneity of effect size regarding the path coefficients of the model. In published MA studies, researchers spend the first half of the results quantifying and detecting the effect size heterogeneity in bivariate relationships and accounting for this heterogeneity using methodological moderators. However, in the second half of the analysis, where a multivariate is employed, the technique entails the assumption that the bivariate relations are drawn from a population, including zero effect size heterogeneity. This fact indicates that MA effect sizes and path estimates may be solely generalizable to a small portion of the population. Yu et al. (2016) and Cheung (2018) dealt with this problem and developed a set of techniques showing the variability surrounding relations in estimating model parameters (i.e., the heterogeneity of effect size) to calibrate the stability of parameters estimates across the population. Using effect size heterogeneity into MASEM would enable researchers to better test and build theory. In this regard, even if a robust statistical test reveals that confidence intervals of path coefficients are significant, it is also necessary to know how those estimates, in the population, are distributed.

We followed Yu et al.’s (2016) and Cheung’s (2018) combined guidelines to ensure the generalizability of findings. Yu et al.’s (2016) methods, namely, Full Information MASEM (FIMASEM), believe that population parameters are assumed to be random variables, including means and standard deviations.

Once effect sizes and their heterogeneity have been combined, the second stage is to apply those combined matrices as the basis to construct a random sample of matrices. It performed in FIMASEM by bootstrapping matrices relying on each effect size and its heterogeneity, namely Standard Deviation (SD). In this method, after the matrices have been produced, the Structural Equation Modeling (SEM) is calculated for each bootstrapped matrix. This step leads to distribution for each estimate (e.g., path coefficient) in the model. Concerning path coefficients, Yu et al. (2016) recommended summarizing the distribution by building Credibility Intervals (CVs) of each path coefficient. Consistent with CVs in the bivariate meta-analysis, CVs indicate the range within which a percentage of the population parameters fall (Yu et al., 2016) suggest constructing 80% CVs). Wider CVs widths imply greater variability estimated parameters in a model (Whitener, 1990).

Despite the advantages of the method introduced by Yu et al. (2016), Cheung (2018) warns some technical errors which Yu et al. (2016) has in the R-coding and suggests corrected ones. According to his re-calculations, the author notes that if researchers aim to identify the effects of heterogeneity concerning the parameter estimates, they may apply the bootstrap CVs proposed by Yu et al.(2016).

However, caution needs to be exercised to use neither the chi-square test nor any cut-offs to assess the generalizability of the proposed model. Hence, we tested the final integrated model based on FIMASEM R-coding presented by Cheung (2018) and established the means of path-coefficients but not goodness-of-fit indices to estimate the generalizability of path coefficients by the width of CVs.

We re-analyzed the model applying FIMASEM with 10,000 bootstrap samples relying on harmonic sample size. We then estimated average path coefficients and standard deviations, and 80% CVs widths for each path coefficient.

This study also used parameter-based MASEM introduced by Cheung et al. (2016, 2018) for the estimation of I^2^ for path coefficients. Cheung et al.(2016) stated that, in the literature, most models and applications of MASEM are correlation-based (Becker, 2009; 1992; Cheung, 2014; 2005; Furlow & Beretvas, 2005; Viswesvaran & Ones, 1995). Correlation-based MASEM follows the random-effects model on the correlation matrices in the first step of the analysis, whereas the estimated pooled correlation matrix is applied to fit structural models in the second stage of the analysis (e.g., Becker, 2009, 1992; Cheung, 2014, 2015). Because MASEM is based on the correlation matrices, it is called correlation-based MASEM.

To illustrate the limitation of correlation-based MASEM, Cheung et al.’s (2016) argued that when the proposed model is an over-identified model, such as SEM, there is no guarantee that the proposed structural model will also fit well in the population correlation vectors of the individual studies even though the proposed model fits well in the average population correlation matrix. Cheung et al. (2016) pointed out that a less conventional approach is to view the parameter estimates as the effect sizes (e.g., Hafdahl, 2009).

This approach is termed as a parameter-based MASEM because meta-analysis is employed to the parameter estimates rather than to the correlation coefficients. Likewise, two stages are involved in parameter-based MASEM. In stage one, the essential assumption of this procedure is that the same structural model exists across all studies, whereas the parameters might differ across studies. In the first stage, the proposed model is fitted to each study. The asymptotic sampling covariance matrix and estimated parameters are available after fitting the model. In the second stage, a multivariate meta-analysis is performed on the effect sizes.

Compared with the correlation-based MASEM, the parameter-based MASEM identifies how the direct effect and the indirect effect vary across studies rather than in how the correlation coefficients vary across studies. Parameter-based MASEM calculates the heterogeneity of the parameter estimates across studies. This method can yield a more nuanced understanding to assess how stable the effect of each predictor is for a regression model. It is challenging to address these types of research questions based on correlation-based MASEM because the variance component of the correlation coefficients is only accessible. Cheung et al. (2018, 2016) noted that parameter-based MASEM could fail when there exist missing effect sizes. The presence of missing effect sizes makes this method difficult to apply because the structural model is fitted for each study. The predictor, mediator, and the dependent variable is necessary to determine the indirect and direct effects in a mediation model. If any of these are not reported, we fail to estimate the expected effect sizes. The current study enabled us to performed parameter-based MASEM to quantify I^2^ of direct and indirect effect and standard deviation of path coefficients due to the lack of missing effect sizes.

We conducted parameter-based MASEM based on R-coding presented by Cheung (2016) to estimates the standardized indirect and direct effects and quantify I^2^ for the direct and indirect effect. In this approach, the delta method was used to calculate the asymptotic covariance matrix. We also calculated the heterogeneity (SD) of the path coefficient estimated by Two-Stage meta-analytic Structural Equation Modeling (TSSEM) relying on R-coding presented by Cheung (2018). The heterogeneity (SD) of the estimated parameter of the TSSEM was calibrated using the bootstrap method. In this method, random correlation matrices were sampled from the TSSEM-Stage one by the parametric bootstrap. The bootstrap method was based on the discussion in Cheung (2018) and Yu et al. (2016). Accordingly, when I^2^ and large-width CVs for each path coefficient values reveal the existence of heterogeneity, inconsistencies were explained by categorical moderator analyses through TSSEM and One Stage Meta-Analytic Structural Equation Modeling (OSMASEM), and also continuous moderator analyses through OSMASEM.

**Supplementary Material H. Sensitivity analyses: checking the robustness of the results**

Outliers were detected by calculating studentized deleted residuals (SDRs) for each primary study (Viechtbauer & Cheung, 2010). SDRs describe the deviation of the correlation of a single sample from the weighted mean correlation of all other involved samples. Studies with SDRs below −1.96 and above +1.96 were considered as outliers.

We also performed TSSEM on corrected correlation matrices, yet compared them for path coefficients with and without these corrections to test the sensitivity of our findings to measurement errors (unreliability corrections). In this regard, the first stage of TSSEM was done through correlation coefficients, which were individually corrected. For each correlation, the individual correction was performed, relying on Hunter and Schmidt’s (2015) approach, that each observed correlation was divided by the square root of the reliability of both variables (Cohen et al.,1983). In some cases, we had to combine several effect sizes and coin an aggregated effect size. To estimate the reliability of these cases, we used a sample size weighted mean reliability. Likewise, we applied a sample size weighted mean reliability for samples where reliability was not reported (Muchinsky, 1996).

Among the 85 samples included in the MASEM analysis, 11 samples did not report reliability for effective parenting, 8 for self-control, 13 for anti-social behaviors. We used means α 0.75, 0.77, and 0.77 for them, respectively. For better comparison between TSSEM with and without corrected correlation, all of the procedures are similar except that corrected correlations were pooled to generate correlation matrix.

We assessed the robustness of the results by comparing the results of TSSEM with other approaches through which MASEM can be conducted (i.e., univariate-r MASEM, FIMASEM, and OSMASEM). The univariate-r MASEM method introduced by Viswesvaran and Ones (1995) mixes Hunter and Schmidt’s (2015) approach and the SEM method. The first stage of the univariate analysis is carried out by pooling the corrected correlation coefficients. For this purpose, we implemented the individual correction for each correlation based on Hunter and Schmidt’s (2015) procedure that was previously noted. The individual corrected correlations incorporated into the meta-analysis show different sample sizes. However, a single sample size for the entire correlation matrix is necessary for analyzing the univariate-r approach MASEM. We exercised a conservative approach, the harmonic mean sample size (n=94604), to fit SEM on the average correlation matrix. We used R-coding presented by Cheung (2019) to implement this method. As pointed out in the previous sections, FIMASEM and OSMASEM are approaches that enable us to perform MASEM. We also compared path coefficients and 95% Confidence Intervals (95%-CIs) estimated by FIMASEM and OSMASEM to check the robustness of the TSSEM results. We used the model for OSMASEM without any moderator. All of the R-codes are available on request from the first author.

**Supplementary Material I. Publication bias**

The current research adds multiple methods of assessing for publication bias, namely, the funnel plot, trim and fill method (Duval & Tweedie, 2000), file drawer analysis (Rosenthal, 1979), and Egger's linear regression test (Egger et al., 1997) to identify the robustness of findings and probable small research effect for single correlations. Funnel plot essentially targets small study bias, in which small studies tend to present larger estimates of effects and higher variability than larger studies. The shape of the scatter plot, in the absence of missing studies, should resemble a symmetrical inverted funnel with a wide base (involving of small studies with large effect estimate variability) and a narrow top (including large studies with small effect estimate variability) (Sterne et al., 2011). The larger and more precision studies regularly cluster around the top of the plot, while smaller studies are typically spread across the bottom of the plot. It is worth noting that presence asymmetry in the funnel plot shows publication bias. In other words, the closer the plot is to a pyramid shape, the lower the publication bias. An ideal funnel plot is one where the involved primary investigations have scattered either side of the pooled effect line in a symmetrical manner. However, severe asymmetry to either side indicates the possibility of publication bias. We adopted the trim and fill to estimate the symmetry of the effect size distributions in the funnel plot. This analysis evaluates the number of missing investigations in light of the most extreme findings in the meta-analysis and also recalculates the estimated effect size to create the funnel plot more symmetrical. Publication bias exists when the estimated effect size varies significantly after applying the trim and fill method. The Rosenthal method (sometimes named a 'file drawer analysis') estimates the number of investigations averaging null findings that would have to be added to the given set of observed findings to decrease the combined significance level (p-value) to an intended alpha level (0.05) (Rosenthal, 1979). In this respect, the fail-safe N value calculates the number of missing primary investigations with null findings that could likely render association non-significant (Hunter & Schmidt, 2015; Orwin, 1983). The high fail-safe N gives confidence in the robustness of findings regarding the possible missing investigations. We also used Egger's linear regression tests. It evaluates the asymmetry of the funnel plots for each effect size. When the statistical p-value is less than 0.05, it means that there exists publication bias in the meta-analysis. It is noteworthy that the research of Lin et al (2018) concluded that concerning the relatively low agreement between different sensitivity and publication bias analyses, meta-analyses should not rely exclusively on a single test, and it is preferable to employ multiple tests with various assumptions. We used the R packages metaSEM to perform MASEM (Cheung, 2019), and the metafor package to assess publication bias, outlier, and influential analyses (Viechtbauer & Cheung, 2010). In this research, the target p-value was equal to 0.05. If the 95% Confidence Intervals (95%-CIs) included zero, we concluded that the intended effect size is non-significant. Correlation based effect sizes were interpreted as small for r < 0.23, medium for r = 0.24 to 0.36, and large for r > 0.37) (Cohen, 1992).

**Supplementary Material J: References to studies used in this meta-analysis**

Anderson, S., Donlan, A. E., McDermott, E. R., & Zaff, J. F. (2015). Ecology matters: Neighborhood differences in the protective role of self-control and social support for adolescent antisocial behavior. *American journal of orthopsychiatry, 85*(6), 536.

Baker, J. O. (2010). The expression of low self-control as problematic drinking in adolescents: An integrated control perspective. *Journal of Criminal Justice, 38*(3), 237-244.

Beaver, K. M. (2008). Nonshared environmental influences on adolescent delinquent involvement and adult criminal behavior. *Criminology, 46*(2), 341-369.

Bobbio, A., Arbach, K., & Vazsonyi, A. T. (2019). Self-control and deviance: a test of the general theory of crime in Argentina. *Victims & Offenders, 14*(1), 119-142.

Boccio, C. M., & Beaver, K. M. (2018). The influence of psychopathic personality traits, low self-control, and nonshared environmental factors on criminal involvement. *Youth Violence and Juvenile Justice, 16*(1), 37-52.

Brauer, J. R. (2011). Autonomy-supportive Parenting and Adolescent Delinquency.

Brownfield, D. (2010). Social control, self-control, and gang membership. *Journal of Gang Research, 17*(4), 1-12.

Burt, C. H., & Ronald, L. (2006). Simons, and Leslie G. Simons. 2006.“A longitudinal test of the effects of parenting and the stability of self-control: Negative evidence for the general theory of crime.”. *Criminology, 44*(2), 353-396.

Burton Jr, V. S., Cullen, F. T., Evans, T. D., Alarid, L. F., & Dunaway, R. G. (1998). Gender, self-control, and crime. *Journal of Research in Crime and Delinquency, 35*(2), 123-147.

Chae, Y. J. (2016). *Unpacking the Black Box of Criminality and Desistance: Self-control and Goal-directed Decision-making.* Northeastern University,

Chen, X. (2017). Parental migration, caretaking arrangement, and children’s delinquent behavior in rural China. *Asian Journal of Criminology, 12*(4), 281-302.

Cheung, M. W. L., & Cheung, S. F. (2016). Random‐effects models for meta‐analytic structural equation modeling: review, issues, and illustrations. *Research synthesis methods, 7*(2), 140-155.

Cheung, N. W., & Cheung, Y. W. (2008). Self-control, social factors, and delinquency: A test of the general theory of crime among adolescents in Hong Kong. *Journal of Youth and Adolescence, 37*(4), 412-430.

Cheung, N. W. T., & Cheung, Y. W. (2010). Strain, self-control, and gender differences in delinquency among Chinese adolescents: Extending general strain theory. *Sociological Perspectives, 53*(3), 321-345.

Cho, S., Hong, J. S., Sterzing, P. R., & Woo, Y. (2005). Parental attachment and bullying in South Korean adolescents: Mediating effects of low self-control, deviant peer associations, and delinquency. *Crime & Delinquency, 63*(9), 1168-1188.

Costello, B. J., & Dunaway, R. G. (2003). Egotism and delinquent behavior. *Journal of Interpersonal Violence, 18*(5), 572-590.

Evans, S. Z., Simons, L. G., & Simons, R. L. (2012). The effect of corporal punishment and verbal abuse on delinquency: Mediating mechanisms. *Journal of Youth and Adolescence, 41*(8), 1095-1110.

Finkenauer, C., Engels, R., & Baumeister, R. (2005). Parenting behaviour and adolescent behavioural and emotional problems: The role of self-control. *International Journal of Behavioral Development, 29*(1), 58-69.

Frijns, T., Finkenauer, C., Vermulst, A. A., & Engels, R. C. (2005). Keeping secrets from parents: Longitudinal associations of secrecy in adolescence. *Journal of Youth and Adolescence, 34*(2), 137-148.

Guo, S. (2018). A model of religious involvement, family processes, self-control, and juvenile delinquency in two-parent families. *Journal of adolescence, 63*, 175-190.

Hay, C. (2001). Parenting, self‐control, and delinquency: A test of self‐control theory. *Criminology, 39*(3), 707-736.

Hay, C., & Forrest, W. (2008). Self‐control theory and the concept of opportunity: The case for a more systematic union. *Criminology, 46*(4), 1039-1072.

Higgins, G. E. (2002). General theory of crime and deviance: A structural equation modeling approach. *Journal of Crime and Justice, 25*(2), 71-95.

Huang, L. (2007). *Family Processes, Low Self-Control, and Deviance: A Longitudinal Test of Self-Control Theory.*

Intravia, J., Jones, S., & Piquero, A. R. (2012). The roles of social bonds, personality, and perceived costs: An empirical investigation into Hirschi’s “new” control theory. *International journal of offender therapy and comparative criminology, 56*(8), 1182-1200.

Janssen, H. J., Eichelsheim, V. I., Deković, M., & Bruinsma, G. J. (2016). How is parenting related to adolescent delinquency? A between-and within-person analysis of the mediating role of self-control, delinquent attitudes, peer delinquency, and time spent in criminogenic settings. *European Journal of Criminology, 13*(2), 169-194.

Janssen, H. J., Eichelsheim, V. I., Deković, M., & Bruinsma, G. J. (2017). Sex differences in longitudinal pathways from parenting to delinquency. *European Journal on Criminal Policy and Research, 23*(4), 503-521.

Jennings, W. G., Higgins, G. E., Tewksbury, R., Gover, A. R., & Piquero, A. R. (2010). A longitudinal assessment of the victim-offender overlap. *Journal of Interpersonal Violence, 25*(12), 2147-2174.

Jeon, H. S., & Chun, J. (2017). The influence of stress on juvenile delinquency: focusing on the buffering effects of protective factors among Korean adolescents. *Social work in public health, 32*(4), 223-237.

Jo, Y., & Lee, B. (2018). Self-control, risky lifestyles, and violent victimization: A longitudinal study of the youth in South Korea. *International Journal of Law, Crime and Justice, 55*, 27-39.

Junger, M., & Tremblay, R. E. (1999). Self-control, accidents, and crime. *Criminal Justice and Behavior, 26*(4), 485-501.

Kabiri, S., Shadmanfaat, S. M., & Donner, C. M. (2019). Examining the Effect of Ineffective Parenting and Low Self-Control on Athletes’ PED Use. *International Criminal Justice Review*, 1057567719832354.

Kazemian, L., Farrington, D. P., & Le Blanc, M. (2009). Can we make accurate long-term predictions about patterns of de-escalation in offending behavior? *Journal of Youth and Adolescence, 38*(3), 384-400.

Kort-Butler, L. A., Tyler, K. A., & Melander, L. A. (2011). Childhood maltreatment, parental monitoring, and self-control among homeless young adults: Consequences for negative social outcomes. *Criminal Justice and Behavior, 38*(12), 1244-1264.

Kuhn, E. S., & Laird, R. D. (2013). Parent and peer restrictions of opportunities attenuate the link between low self‐control and antisocial behavior. *Social Development, 22*(4), 813-830.

Li, J.-B., Liberska, H., Salcuni, S., & Delvecchio, E. (2019). Aggressive perpetration and victimization among Polish male and female adolescents: The role of attachment to parents and self-control. *Crime & Delinquency, 65*(3), 401-421.

Liu, L., Wang, N., & Tian, L. (2019). The parent-adolescent relationship and risk-taking behaviors among Chinese adolescents: The moderating role of self-control. *Frontiers in Psychology, 10*.

Longshore, D., Chang, E., & Messina, N. (2005). Self-control and social bonds: A combined control perspective on juvenile offending. *Journal of Quantitative Criminology, 21*(4), 419-437.

Marcotte, G., Marcotte, D., & Bouffard, T. (2002). The influence of familial support and dysfunctional attitudes on depression and delinquency in an adolescent population. *European Journal of Psychology of Education, 17*(4), 363.

McGloin, J. M., Pratt, T. C., & Maahs, J. (2004). Rethinking the IQ-delinquency relationship: A longitudinal analysis of multiple theoretical models. *Justice Quarterly, 21*(3), 603-635.

McKee, J. R. (2012). The moderation effects of family structure and low self-control. *American Journal of Criminal Justice, 37*(3), 356-377.

Meldrum, R. C., Barnes, J., & Hay, C. (2013). Sleep deprivation, low self-control, and delinquency: A test of the strength model of self-control. *Journal of Youth and Adolescence, 44*(2), 465-477.

Meldrum, R. C., Young, J. T., & Lehmann, P. S. (2015). Parental low self-control, parental socialization, young adult low self-control, and offending: A retrospective study. *Criminal Justice and Behavior, 42*(11), 1183-1199.

Meldrum, R. C., Young, J. T., & Weerman, F. M. (2009). Reconsidering the effect of self-control and delinquent peers: Implications of measurement for theoretical significance. *Journal of Research in Crime and Delinquency, 46*(3), 353-376.

Miller, H. V. (2012). Correlates of delinquency and victimization in a sample of Hispanic youth. *International Criminal Justice Review, 22*(2), 153-170.

Moon, B., & Alarid, L. F. (2015). School bullying, low self-control, and opportunity. *Journal of Interpersonal Violence, 30*(5), 839-856.

Moon, B., & Morash, M. (2013). General strain theory as a basis for the design of school interventions. *Crime & Delinquency, 59*(6), 886-909.

Moon, B., Morash, M., & McCluskey, J. D. (2012). General strain theory and school bullying: An empirical test in South Korea. *Crime & Delinquency, 58*(6), 827-855.

Morris, G. D. (2003). *Cultural Discontinuity, Self-control, and Native American Delinquency.* Mississippi State University. Department of Sociology, Anthropology, and …,

Muftić, L. R., Grubb, J. A., Bouffard, L. A., & Maljević, A. (2014). The impact of life domains on juvenile offending in Bosnia and Herzegovina: Direct, indirect, and moderating effects in Agnew’s integrated general theory. *Journal of Research in Crime and Delinquency, 51*(6), 816-845.

Owens-Sabir, M. C. (2005). *The continuum of control and self-esteem: A study of delinquency*: Mississippi State University.

Özdemir, Y., Vazsonyi, A. T., & Cok, F. (2013). Parenting processes and aggression: The role of self-control among Turkish adolescents. *Journal of adolescence, 36*(1), 65-77.

Schreck, C. J. (2002). How do social bonds restrain crime? A study of the mechanisms. *Journal of Crime and Justice, 25*(2), 1-21.

Schreck, C. J., Wright, R. A., & Miller, J. M. (2002). A study of individual and situational antecedents of violent victimization. *Justice Quarterly, 19*(1), 159-180.

Shadmanfaat, S. M. S., Howell, C. J., Muniz, C. N., Cochran, J. K., Kabiri, S., & Richardson, D. A. (2018). The Predictive Ability of Self-Control and Differential Association on Sports Fans' Decision to Engage in Cyber Bullying Perpetration against Rivals. *International Journal of Cyber Criminology, 12*(2), 362-375.

Simons, R. L., Simons, L. G., CHEN, Y. F., Brody, G. H., & LIN, K. H. (2007). Identifying the psychological factors that mediate the association between parenting practices and delinquency. *Criminology, 45*(3), 481-517.

Toro, R. I. (2010). *Acculturation gaps in Latino families: Prospective family mediators associated with child outcomes.* UC Riverside,

Unnever, J. D., Cullen, F. T., & Agnew, R. (2006). Why is “bad” parenting criminogenic? Implications from rival theories. *Youth Violence and Juvenile Justice, 4*(1), 3-33.

van Gelder, J.-L., Averdijk, M., Ribeaud, D., & Eisner, M. (2017). Punitive parenting and delinquency: The mediating role of short-term mindsets. *The British Journal of Criminology, 58*(3), 644-666.

Vazsonyi, A. T., & Belliston, L. M. (2007). The family→ low self-control→ deviance: A cross-cultural and cross-national test of self-control theory. *Criminal Justice and Behavior, 34*(4), 505-530.

Vazsonyi, A. T., & Huang, L. (2010). Where self-control comes from: On the development of self-control and its relationship to deviance over time. *Developmental psychology, 46*(1), 245.

Vazsonyi, A. T., Jiskrova, G. K., Ksinan, A. J., & Blatný, M. (2016). An empirical test of self-control theory in Roma adolescents. *Journal of Criminal Justice, 44*, 66-76.

Vazsonyi, A. T., & Klanjšek, R. (2008). A test of self‐control theory across different socioeconomic strata. *Justice Quarterly, 25*(1), 101-131.

Vera, E. P., & Moon, B. (2013). An empirical test of low self-control theory: Among Hispanic youth. *Youth Violence and Juvenile Justice, 11*(1), 79-93.

Watts, S. J., & McNulty, T. L. (2016). Genes, parenting, self-control, and criminal behavior. *International journal of offender therapy and comparative criminology, 60*(4), 469-491.

Wells, J., Armstrong, T., Boutwell, B., Boisvert, D., Flores, S., Symonds, M., & Gangitano, D. (2015). Molecular genetic underpinnings of self-control: 5-HTTLPR and self-control in a sample of inmates. *Journal of Criminal Justice, 43*(5), 386-396.

Woeckener, M., Boisvert, D. L., Cooke, E. M., Kavish, N., Lewis, R. H., Wells, J., . . . Harper, J. M. (2018). Parental rejection and antisocial behavior: the moderating role of testosterone. *Journal of criminal psychology, 8*(4), 302-313.

Wright, B. R. E., Caspi, A., Moffitt, T. E., & Silva, P. A. (2001). The effects of social ties on crime vary by criminal propensity: A life‐course model of interdependence. *Criminology, 39*(2), 321-348.

You, S., & Kim, A. Y. (2016). Understanding aggression through attachment and social emotional competence in Korean middle school students. *School psychology international, 37*(3), 255-270.

**Supplementary Material K: Additional references**

Alexander, R. A. (1990). A note on averaging correlations. *Bulletin of the Psychonomic Society, 28*(4), 335-336.

Alvarez-Rivera, L. L., & Fox, K. A. (2010). Instittutional attachments and self-control: Understanding deviance among Hispanic adolescents. *Journal of Criminal Justice, 38*(4), 666-674.

Becker, B. (2009). Model-based MASEM. *The handbook of research synthesis and meta-analysis*, 377-395.

Becker, B. J. (1992). Using results from replicated studies to estimate linear models. *Journal of Educational Statistics, 17*(4), 341-362.

Cheung, M. W.-L. (2014). Fixed-and random-effects meta-analytic structural equation modeling: Examples and analyses in R. *Behavior research methods, 46*(1), 29-40.

Cheung, M. W.-L. (2015). *Meta-analysis: A structural equation modeling approach*: John Wiley & Sons.

Cheung, M. W.-L. (2018). Issues in solving the problem of effect size heterogeneity in meta-analytic structural equation modeling: A commentary and simulation study on Yu, Downes, Carter, and O’Boyle (2016).

Cheung, M. W.-L. (2019). metaSEM: An R package for meta-analysis using structural equation modeling. *Frontiers in Psychology, 5*, 1521.

Cheung, M. W.-L., & Chan, W. (2005). Meta-analytic structural equation modeling: a two-stage approach. *Psychological methods, 10*(1), 40.

Cho, S., Glassner, S., & Lee, J. M. (2019). Impact of low self-control, parental involvement, and peer relationships on changes of bullying perpetration over time: A latent growth curve model of a sample of South Korean adolescents. *Children and Youth Services Review*, 104397.

Cohen, J., Cohen, P., West, S. G., & Aiken, L. S. (1983). Applied multiple regression. *Correlation Analysis for the Behavioral Sciences, 2*.

Duval, S., & Tweedie, R. (2000). A nonparametric “trim and fill” method of accounting for publication bias in meta-analysis. *Journal of the American Statistical Association, 95*(449), 89-98.

Egger, M., Smith, G. D., Schneider, M., & Minder, C. (1997). Bias in meta-analysis detected by a simple, graphical test. *Bmj, 315*(7109), 629-634.

Eid, M., Gollwitzer, M., & Schmitt, M. (2010). Statistik und Forschungsmethoden. In.

Furlow, C. F., & Beretvas, S. N. (2005). Meta-analytic methods of pooling correlation matrices for structural equation modeling under different patterns of missing data. *Psychological Methods, 10*(2), 227.

Hafdahl, A. (2009). Meta-analysis for functions of heterogeneous multivariate effect sizes.

Higgins, J. P. (2008). Commentary: Heterogeneity in meta-analysis should be expected and appropriately quantified. *International journal of epidemiology, 37*(5), 1158-1160.

Hunter, J. E., & Schmidt, F. L. (2015). *Methods of meta-analysis: Correcting error and bias in research findings*: Sage.

Lin, L., & Chu, H. (2018). Quantifying publication bias in meta‐analysis. *Biometrics, 74*(3), 785-794.

Meldrum, R. C. (2008). Beyond parenting: An examination of the etiology of self-control. *Journal of Criminal Justice, 36*(3), 244-251.

Muchinsky, P. M. (1996). The correction for attenuation. *Educational and psychological measurement, 56*(1), 63-75.

Olkin, I., & Pratt, J. W. (1958). Unbiased estimation of certain correlation coefficients. *The Annals of Mathematical Statistics, 29*(1), 201-211.

Orwin, R. G. (1983). A fail-safe N for effect size in meta-analysis. *Journal of educational statistics, 8*(2), 157-159.

Rosenthal, R. (1979). The file drawer problem and tolerance for null results. *Psychological bulletin, 86*(3), 638.

Viechtbauer, W., & Cheung, M. W. L. (2010). Outlier and influence diagnostics for meta‐analysis. *Research synthesis methods, 1*(2), 112-125.

Viswesvaran, C., & Ones, D. S. (1995). Theory testing: Combining psychometric meta‐analysis and structural equations modeling. *Personnel psychology, 48*(4), 865-885.

Whitener, E. M. (1990). Confusion of confidence intervals and credibility intervals in meta-analysis. *Journal of Applied Psychology, 75*(3), 315.

Yu, J. J., Downes, P. E., Carter, K. M., & O'Boyle, E. H. (2016). The problem of effect size heterogeneity in meta-analytic structural equation modeling. *Journal of Applied Psychology, 101*(10), 1457.
